# Deep Transfer Learning Links Benign Glands to Prostate Cancer Progression via Transcriptomics

**DOI:** 10.1093/gpbjnl/qzaf119

**Published:** 2025-11-29

**Authors:** Justin L Couetil, Ziyu Liu, Chao Chen, Ahmed K Alomari, Kun Huang, Jie Zhang, Travis S Johnson

**Affiliations:** Department of Medical and Molecular Genetics, IU School of Medicine, Indianapolis, IN 46202, USA; Indiana Clinical and Translational Sciences Institute, Indianapolis, IN 46202, USA; Department of Statistics, Purdue University, West Lafayette, IN 47907, USA; Department of Biomedical Informatics, Stony Brook University, Stony Brook, NY 11794, USA; Department of Medical and Molecular Genetics, IU School of Medicine, Indianapolis, IN 46202, USA; Department of Pathology, IU School of Medicine, Indianapolis, IN 46202, USA; Department of Medical and Molecular Genetics, IU School of Medicine, Indianapolis, IN 46202, USA; Department of Biostatistics and Health Data Sciences, IU School of Medicine, Indianapolis, IN 46202, USA; Melvin and Bren Simon Comprehensive Cancer Center, Indianapolis, IN 46202, USA; Center for Computational Biology and Bioinformatics, IU School of Medicine, Indianapolis, IN 46202, USA; Department of Medical and Molecular Genetics, IU School of Medicine, Indianapolis, IN 46202, USA; Melvin and Bren Simon Comprehensive Cancer Center, Indianapolis, IN 46202, USA; Center for Computational Biology and Bioinformatics, IU School of Medicine, Indianapolis, IN 46202, USA; Department of Biostatistics and Health Data Sciences, IU School of Medicine, Indianapolis, IN 46202, USA; Melvin and Bren Simon Comprehensive Cancer Center, Indianapolis, IN 46202, USA; Center for Computational Biology and Bioinformatics, IU School of Medicine, Indianapolis, IN 46202, USA; Indiana Biosciences Research Institute, Indianapolis, IN 46202, USA

**Keywords:** Spatial transcriptomics, Single-cell transcriptomics, Prostate cancer, Deep learning, Tumor immune microenvironment

## Abstract

The field effect describes the phenomena where environmental exposures, infection, and genetic predisposition result in molecular changes in cells that predispose them to developing cancer. Though this is a well-established concept in pathology, it remains underexplored in the context of high-resolution omics. We utilized the Diagnostic Evidence Gauge of Single Cells (DEGAS) deep transfer learning framework to analyze prostate cancer spatial transcriptomics to identify cells and tissues that are highly associated with cancer progression. DEGAS highlighted morphologically benign glands with reduced expression of *microseminoprotein-beta* (*MSMB*), a differentiation marker downregulated in aggressive tumors. These glands have upregulated genes associated with antigen presentation and aggressive neoplasms. Integration of single-cell transcriptomics and deep learning image analysis separately revealed altered immune-cell infiltration, suggesting a complex interplay in the tumor environment, facilitating aggressiveness. We used immunohistochemistry to quantify the MSMB protein (PSP-94) expression in morphologically normal and tumor tissues from patients with and without 5-year distant metastasis. Samples from patients who developed metastasis consistently showed lower fractions of positively stained cells, indicating a subtle yet significant “field effect” in seemingly benign regions. These proteomic results validate the transcriptomic findings and further underscore that inflammatory or immune-related changes in ostensibly normal tissue may contribute to aggressive disease progression.

## Graphical Abstract

**Graphical Abstract:**
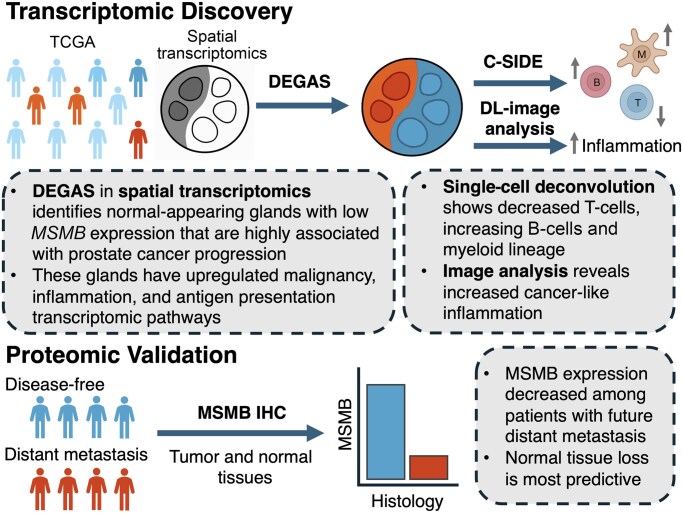

## Introduction

Prostate cancer is the most common malignancy among men in the United States [1]. Fortunately, the 5-year survival rates have improved significantly for localized and regional disease, but it remains the second most common cause of cancer-related deaths [2]. Subsets of patients are much more likely to have a cancer that will spread to distant organs (*i.e.*, metastasis), and there are few tools to identify and treat these patients [3]. Unfortunately, patients with distant metastases have a 5-year survival rate of just 34.1% [1]. Given the high incidence of prostate cancer and the increasing incidence of metastatic cancer patients each year [4], developing technologies to identify high-risk prostate cancer patients is of utmost importance.

Clinicians have few tools to identify patients at high-risk for disease progression. Recently, the United States Preventive Services Task Force has advised against using prostate specific antigen (PSA) as a screening tool for prostate cancer diagnosis [5]. PSA is a highly sensitive biomarker for prostate cancer, but does not discriminate between high and low-risk disease. Notably, multi-center studies have demonstrated that low-risk prostate cancer is often over-treated, making the tradeoff between morbid excisional surgery and uncertain survival benefit unacceptable for many patients [6]. There is a critical need for a reliable biomarker that will identify the subset of prostate cancer that would benefit from intervention [7].

Developing prognostic models for prostate cancer has been difficult due to a high degree of genetic variability [8]. Prostate cancer has high intra- and inter-tumor genomic heterogeneity, and multiple polyclonal tumors can arise simultaneously [9]. Until recently, this heterogeneity has been difficult to study because techniques like bulk omics analyses (*e*.*g*., RNA-seq) and image-based techniques (*e.g.*, immunohistochemistry, IHC) either lack spatial information or are very low throughput with few target molecules per sample. Single cell (SC) analysis then enabled deconvolution (*i*.*e*., decomposition) of the tumor and microenvironment into their constituent cells [10].

SC analysis is limited, however, because it lacks both the spatial information and the morphological information of the cells being analyzed. By the central dogma, identifying cell types based solely on gene expression may obscure cells that are phenotypically normal but transcriptionally abnormal. This is directly relevant to phenomena like the epithelial-to-mesenchyme transition, which is fundamental to cancer progression [11]. Fortunately, high-throughput spatial transcriptomics (ST) has recently become available, allowing for improved study of the heterogeneity of tumors and the microenvironment with a spatial context.

ST has the potential to recognize how diverse components of the tumor and microenvironment collectively contribute to clinical outcomes. SC and ST data have been used to reveal an enormous transcriptomic diversity within and between tumors. However, identifying reliable cell gene markers in these heterogeneous and dynamic systems is a key challenge [12]. Relying on human curated knowledge of gene markers and cell morphology to assign simple labels may obscure transitional cell states of both epithelial [13] and immune cells [14]. Similarly, though ST provides the ability to simultaneously analyze transcriptomics and histopathology, traditional visual assessment by humans is semi-quantitative, and machine learning approaches are needed to detect subtle systematic differences in “normal” tissue associated with clinical outcomes [15].

To address this, we applied the Diagnostic Evidence Gauge of Single Cells (DEGAS) deep transfer learning to ST. This framework has been successfully applied to single-cell transcriptomics to identify clinically relevant molecular features in multiple cancers as well as neurodegenerative diseases [16]. DEGAS integrates bulk sequencing data with clinical outcomes to map these associations onto high-resolution transcriptomic data. It employs domain adaptation losses to align data distributions across different modalities, enabling the identification of transcriptomic signatures associated with disease progression. In this study, we extended DEGAS to ST to dissect the tumor and microenvironment of prostate cancer.

This observed heterogeneity reflects the “field effect”, which has complicated the definition of “normal” tissue for decades [17]. The field effect describes the finding that chronic inflammation, driven by exposures and genetic predisposition, causes histologically normal cells to acquire genetic and epigenetic changes that contribute to the development of cancer [18]. These genetic changes reflect etiology and contribute to cancer progression [19–21]. Aligning with this, applying DEGAS to ST identified inflammatory markers and subtle morphological differences in histologically normal prostate glands that were highly associated with cancer progression. Tools like DEGAS can help in identifying field effects associated with clinical outcomes and, therefore, may be useful for screening and targeted therapies.

## Results

### Study Overview

**Figure 1:**
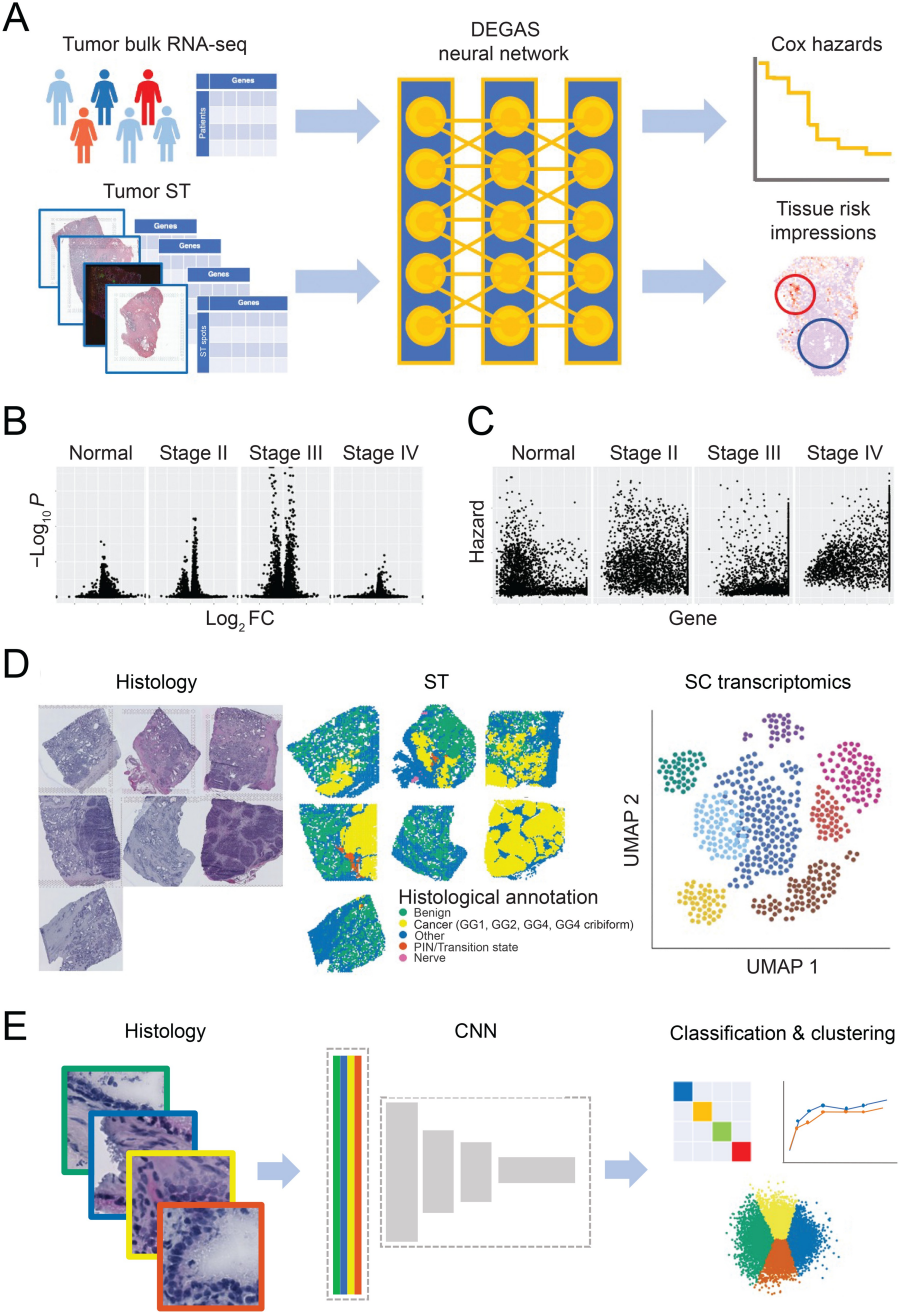
DEGAS workflow **A**. Integration of bulk and spatial transcriptomics. Patient bulk tumor RNA-seq was used to predict clinical outcomes and phenotypic information using a deep-learning framework that simultaneously matches ST gene expression distribution to that of patients. By aligning gene expression distributions across these two data types, DEGAS can then assign (“transfer”) risk scores to the spatial transcriptomic arrays, highlighting high-risk tissue. **B**. Comparison of RNA expression in high and low-risk tissues. **C**. Correlations between gene expression and risk in high and low-risk tissues. **D**. Morphological identification of BGs with malignant transcriptional profiles in an additional dataset. Integration with SC data allows for identification of cell-type specific expression changes associated with poor progression-free survival, and SC clusters annotated as cancer localize to histologically benign regions of ST data. **E**. Deep learning approaches for analyzing the histology from these different regions, identifying similarities between the high-risk glands, high-grade cancer, and inflammation. t-SNE graphic was generated from BioRender. RNA-seq, RNA sequencing; ST, spatial transcriptomics; DEGAS, Diagnostic Evidence Gauge of Single cells; BG, benign gland; SC, single cell; t-SNE, *t*-distributed stochastic neighbor embedding; CNN, convolutional neural network.

**Figure 2:**
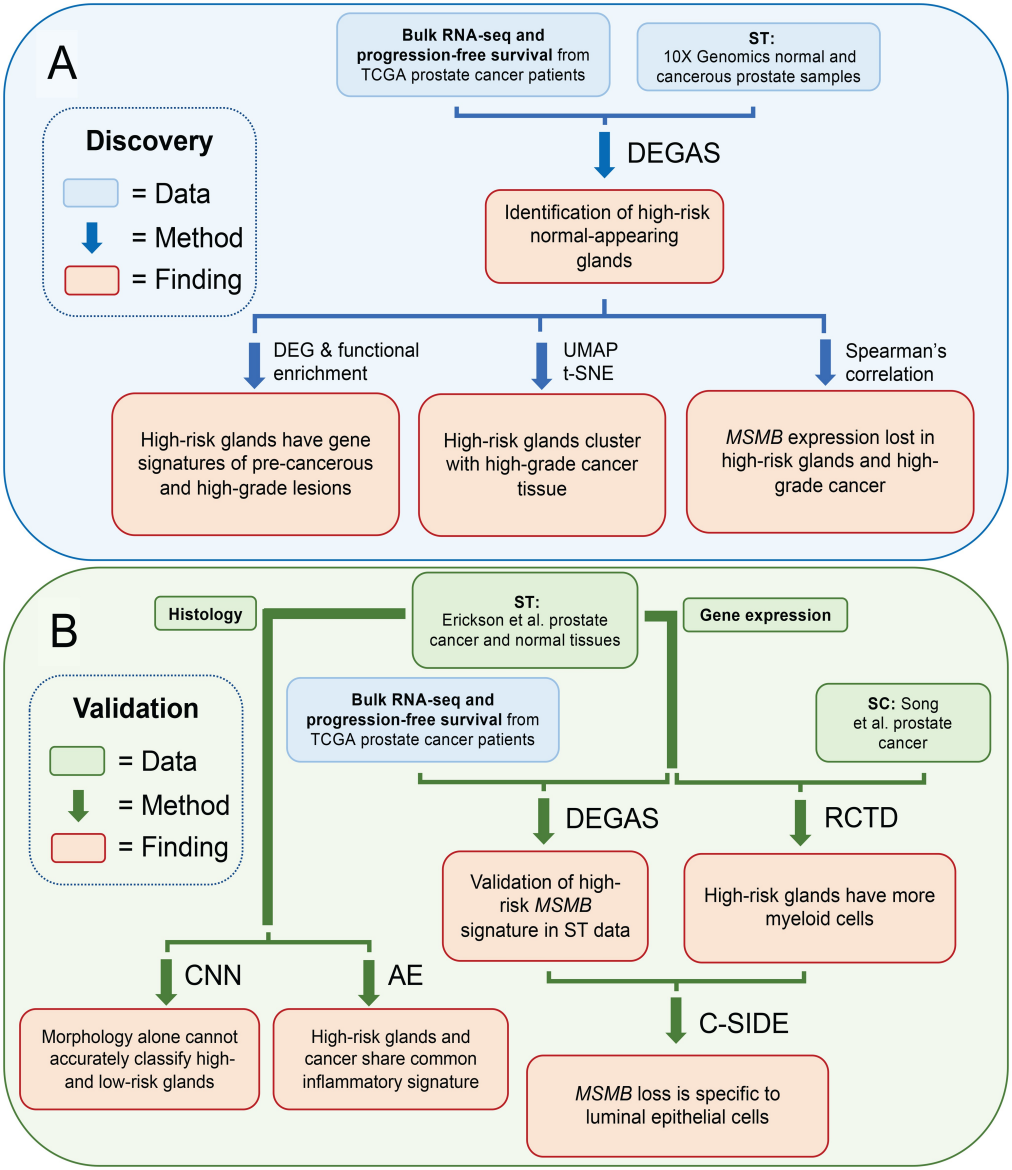
Experimental overview **A**. In the discovery workflow, a DEGAS model is trained to predict progression-free survival from the RNA-seq of prostate cancer TCGA patients. This information is mapped onto four ST samples from 10X Genomics, identifying benign morphology glands that are highly associated with poor progression-free survival. Functional enrichment shows these high-risk glands have signatures of carcinogenesis and high-grade cancer. *MSMB* is consistently negatively correlated with risk. **B**. We validated these findings in seven ST samples from Erickson et al. [28]. Using the tool RCTD, we deconvolved these samples with an SC dataset from Song et al. [29]. This demonstrates that cancer cell clusters are attributed to histologically BGs. C-SIDE of the deconvoluted ST data shows that *MSMB* loss is specific to high-risk luminal epithelial cells, the originators of prostate cancer. Finally, we investigated whether there might be morphologic features that are too subtle for a pathologist to interpret but could be discerned by deep-learning models. The CNN was unable to accurately differentiate the high and low-risk glands. Taking an unsupervised approach, an AE was used to compress images. These compressed representations were clustered, revealing one cluster that was disproportionally represented among the regions of the highest risk glands, highest grade cancer, and inflammation. TCGA, The Cancer Genome Atlas; RCTD, Robust Cell Type Decomposition; C-SIDE, Cell-type Specific Inference of Differentiation Expression; AE, autoencoder; UMAP, uniform manifold approximation and projection; *MSMB*, *microseminoprotein-beta*.

The recently developed tool DEGAS [16] was applied in ST to understand what prostate tissues contribute to aggressive cancers (**Figure 1**), and the transcriptomics (**Figure 2**), cell type composition (Figure 1D), and tissue morphology of these high-risk regions were analyzed (Figure 1E). The overall experimental workflow (Figure 2), results of feature selection (Figure S1), and results of model aggregation (Figure S2) are all outlined. The resources used in this study (**Table 1**) are reported in File S1 in compliance with STROBE guidelines to the extent that metadata were available [22].

**Table 1:** Overview of data sources, sample size, assay type, and additional attributes for prostate cancer datasets used in discovery and validation experiments

Experiment	Dataset	Sample size	Data type	Additional attribute
Discovery & validation	TCGA-PRAD	493 patients	RNA-seq & progression-free survival	
Discovery dataset	10X Genomics	Normal: 2543 spots	10X Visium ST	Histological labels
		Stage II: 3460 spots		
		Stage III: 4371 spots		
		Stage IV: 3043 spots		
Validation dataset	[28]	H1_2: 2775 spots	10X Visium ST	Histological labels & whole-slide images
		H1_4: 4079 spots		
		H1_5: 3856 spots		
		H2_1: 3092 spots		
		H2_5: 3554 spots		
		V1_2: 2736 spots		
	[29]	21,743 cells from prostate cancer and surrounding tissue	Seq-Well SC	Cell type annotations

*Note*: TCGA, The Cancer Genome Atlas; PRAD, prostate adenocarcinoma; RNA-seq, ribonucleic acid sequencing; ST, spatial transcriptomics; SC, single cell.

### Discovery cohort analysis

#### DEGAS identifies morphologically BGs associated with diminished progression-free survival

**Figure 3:**
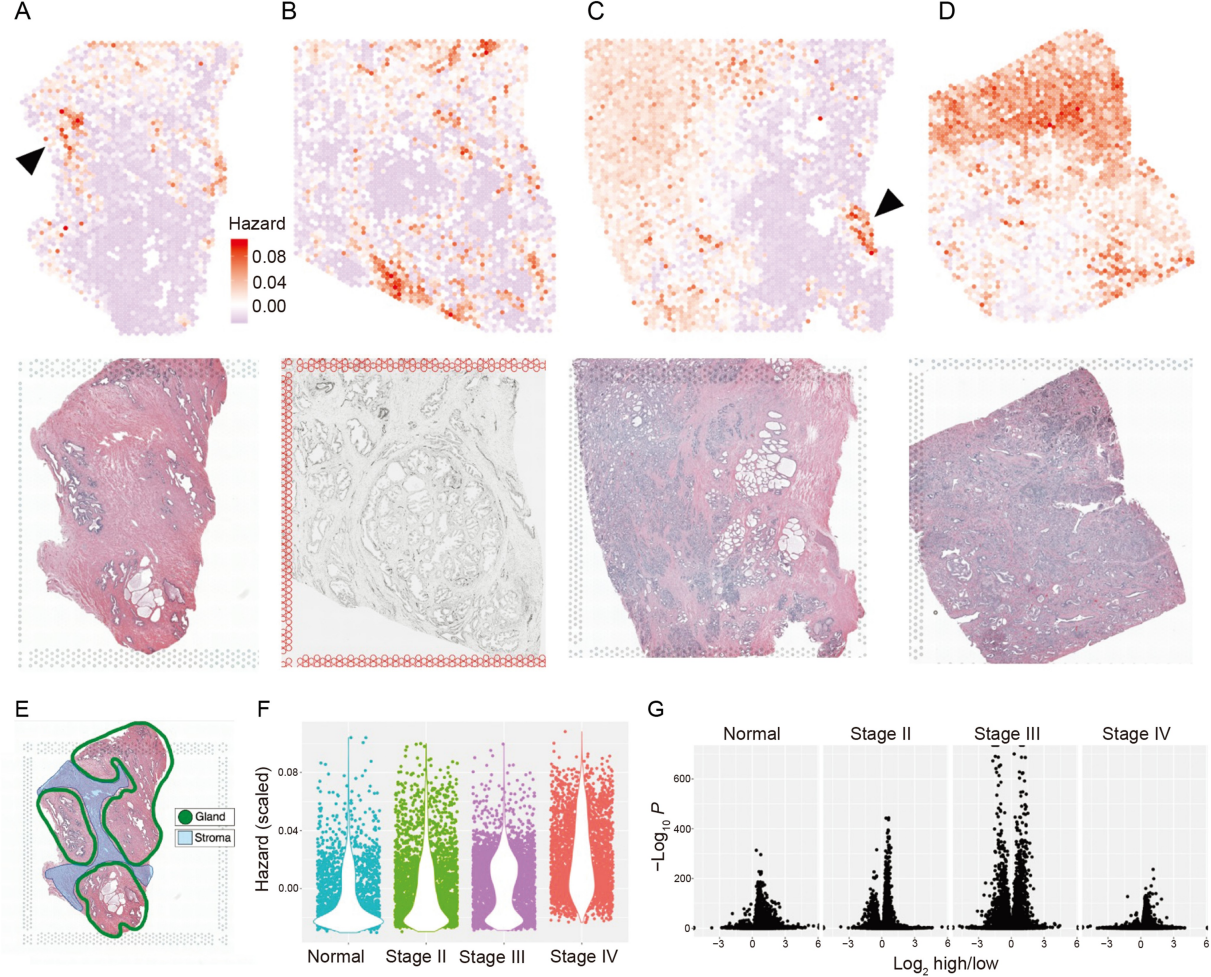
DEGAS risk impressions in four spatial transcriptomic samples of prostate tissue **A**. Histologically normal prostate tissue. The black arrow indicated a particularly high-risk region, with a corresponding H&E-stained section below. **B**. Stage II prostate adenocarcinoma with tissue section below. This specimen was stained with immunofluorescence and thus not H&E-stained. **C**. Stage III prostate adenocarcinoma with right-adjacent normal tissue (blue), and an arrow indicating a particularly high-risk region. In the corresponding H&E, below, examination by a pathologist revealed that the region is a nerve. **D**. Stage IV prostate acinar cell carcinoma (*i.e.*, adenocarcinoma) and corresponding H&E section below. **E**. Histological labels from normal prostate tissue shown in A. **F**. Scatter plot overlaid with violin plot of the hazards for all ST spots in Panels A–D, showing trend of increasing hazard with increasing stage. **G**. Differential gene expression plot using Wilcoxon test FDR-adjusted *P* values, and the log_2_ mean fold change of the samples in panels A–D. High (red) and low (blue) risk scores from the DEGAS model are visualized. All samples share the same hazard/color scale. H&E, Hematoxylin and eosin.

We used our newly established pipeline (Figures 1 and 2) to generate DEGAS risk scores for ST data. DEGAS risk scores highlighted certain tissues in the normal prostate sample that were associated with cancer progression (**Figure 3**A). A comprehensive review of the hematoxylin and eosin (H&E)-stained whole-slide image by a pathologist revealed that the DEGAS heatmap was highlighting glandular tissue. The highest-risk glands (Figure 3A and E) were devoid of any morphological indicators typically associated with malignancy, and no morphological signs of neoplasia were present (Figure S3A). The pathologist noted only a few instances of urothelial metaplasia, a benign phenomenon in which urothelial cells coexist with epithelia of the peripheral prostatic glands and ducts [23].

Histological characteristics associated with DEGAS risk predictions in the prostate cancer tissue samples were annotated. However, identifying correlates was not feasible for the Stage II prostate adenocarcinoma due to the absence of a diagnostic H&E-stained image (Figure 3B). In the Stage III prostate adenocarcinoma (Figure 3C), the high-risk region on the left corresponded with the bulk tumor. Conversely, on the right, both the peritumoral stroma and some prostatic glands were identified as low-risk (highlighted in blue), interspersed with a few high-risk benign glands (BGs, marked in red). Notably, an isolated nerve in the stroma of the Stage III sample was identified as high-risk (Figure 3C). For the Stage IV sample (Figure 3D), the areas identified as high-risk were consistent with regions displaying slightly higher Gleason score morphologies, characterized by closely packed glands, some dissociated cancer cells, and a prominent presence of lymphocytes (Figure S3B). Intuitively, DEGAS risk scores increased with increasing cancer stage (*P* = 8.32E−4, *P* = 8.14E−26, *P* = 1.54E−228; Figure 3F).

#### Differential expression in high-risk normal prostate glands reveals signatures associated with high-grade prostate cancer and carcinogenesis

Differential expression (DE) analysis was used to pinpoint genes that discriminate between high-risk and low-risk regions (Figure 3G). For the normal prostate sample, DE was performed between only the high and low-risk glands, thereby avoiding potential confounders from comparing against other low-risk histologic regions, like the collagenous stroma. Subsequent functional enrichment analysis of DEGs from the normal samples revealed upregulation of RNA signatures associated with pathways of carcinogenesis, congenital genetic disorders, inflammation, immune interactions, and high-grade cancers. Notably, these upregulated pathways include Triple Negative Breast Neoplasms (*P* = 6.53 × 10^−9^), Neoplasm invasiveness (*P* = 9.91 × 10^−9^), Epithelium development (*P* = 7.29 × 10^−7^), and Antigen processing and presentation of exogenous antigen via MHC Class II (*P* = 8.81 × 10^−6^; Table S1). Analysis across the Normal, Stage II, Stage III, and Stage IV samples revealed four common enrichment categories among the upregulated DEGs: High-grade prostatic intraepithelial neoplasia, Hormone refractory prostate cancer, Prostatic neoplasms, and Renal Cell Carcinoma (**Table 2**; Figures S4 and S5).

**Table 2:** Overlap in functional enrichment of high-risk regions in normal, stage II, III, and IV samples

Enrichment term	Gene	Normal	Stage II	Stage III	Stage IV
High-grade prostatic intraepithelial neoplasia	*ACTB*, *GSTP1*, *IGFBP3*, *SQSTM1*, *CTNNB1*	3.78E−03	2.23E−03	4.32E−03	9.49E−03
Hormone refractory prostate cancer	*PSAP*, *HMGB1*, *PABPC1*, *DDX5*, *GSTP1*, *IGFBP3*, *CLU*, *AHNAK*, *SLPI*, *CTNNB1*, *MMP7*, *NONO*, *HSPB1*	9.46E−04	1.65E−04	1.74E−03	3.05E−04
Prostatic neoplasm	*LITAF*, *B2M*, *RUNX1*, *GSTP1*, *IGFBP3*, *ZFP36L2*, *CLU*, *EHF*, *CTNNB1*, *CTSB*, *CTSD*, *CNN3*	1.40E−03	3.47E−05	5.61E−06	3.05E−04
Renal cell carcinoma	*ACTB*, *GSTP1*, *IGFBP3*, *SQSTM1*, *CTNNB1*	2.84E−07	2.16E−11	8.37E−03	4.91E−05

*Note:* ACTB, actin beta; GSTP1, glutathione S-transferase pi 1; IGFBP3, insulin-like growth factor binding protein 3; SQSTM1, sequestosome-1; CTNNB1, catenin beta 1; PSAP, prosaposin; HMGB1, high mobility group box 1; PABPC1, poly(A) binding protein cytoplasmic 1; DDX5, DEAD-box helicase 5; CLU, clusterin; AHNAK, AHNAK nucleoprotein; SLPI, secretory leukocyte peptidase inhibitor; MMP7, matrix metallopeptidase 7; NONO, non-POU domain containing octamer binding; HSPB1, heat shock protein family B (small) member 1; LITAF, lipopolysaccharide induced TNF factor; B2M, beta-2-microglobulin; RUNX1, RUNX family transcription factor 1; ZFP36L2, ZFP36 ring finger protein like 2; EHF, ETS homologous factor; CTSB, cathepsin B; CTSD, cathepsin D; CNN3, calponin 3.

#### Loss of MSMB is strongly associated with high-risk BGs

**Figure 4:**
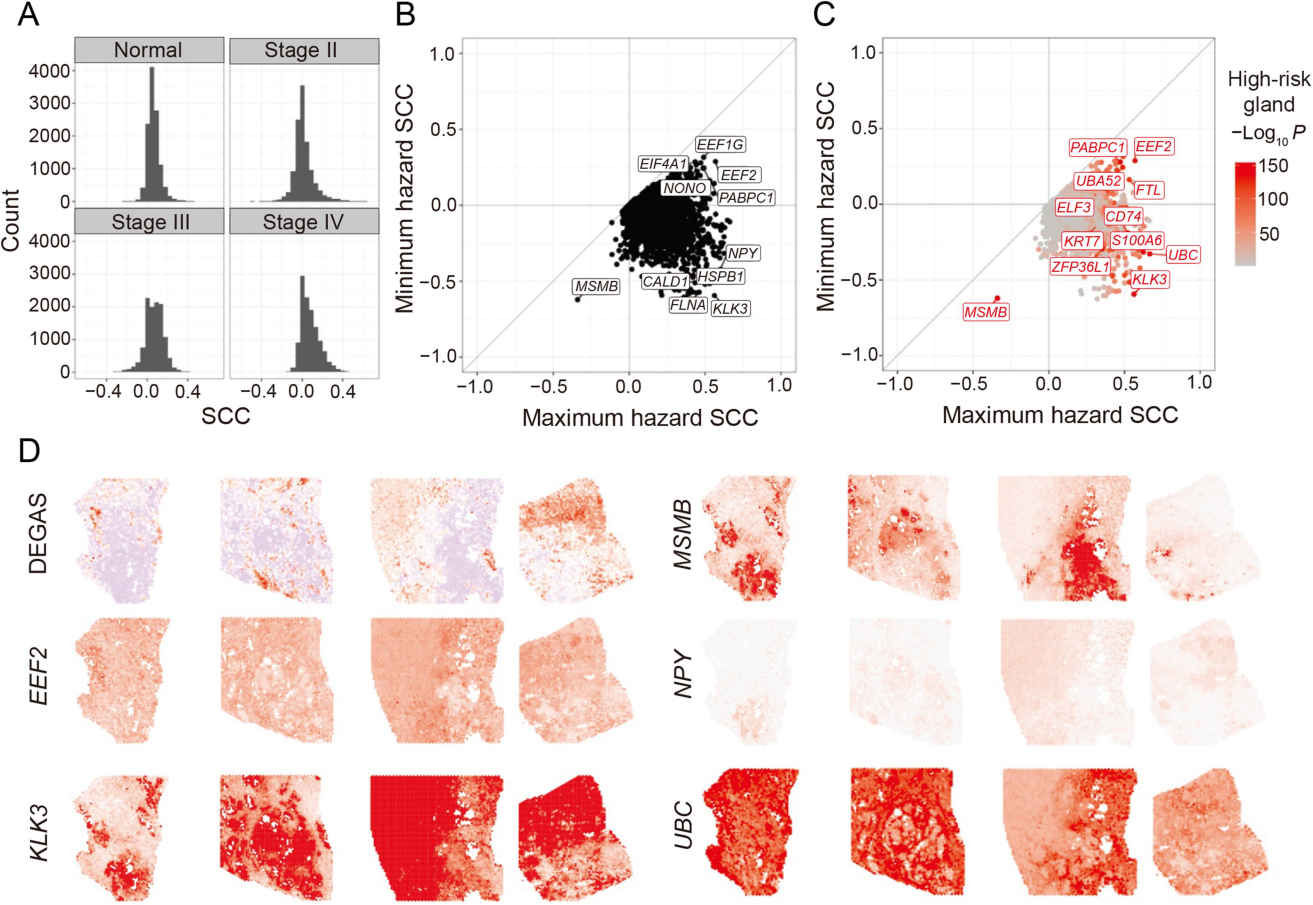
Coefficients of Spearman’s correlation between genes and DEGAS hazard output across all ST spots on all prostate cancer samples **A**. Histogram of correlation coefficients between genes and the DEGAS hazard output for each spot. The distributions are different. For the normal sample, the SCC was not restricted to only glandular tissues (thus, the title refers to “all ST spots”). **B**. Concordance between SCC across the different prostate samples. It is demonstrated by plotting the range of coefficients across the four samples (maximum and minimum). *MSMB* is the only gene for which the SCC is consistently negative across all slides. *EEF2* and others are consistently associated with high hazard. Some genes with positive and negative coefficients are *KLK3*, *NPY*, *HSPB1*, and *CALD1*. **C**. Panel B overlaid with the FDR adjusted −log *P* value for each gene’s enrichment in the high-risk glandular tissues. Labeled genes have −log *P* value > 120. **D**. Expression of representative genes from panels B & C visualized on four prostate ST samples, in order from left to right: Normal, Stage II, Stage III, and Stage IV. DEGAS hazard provided top left for reference, where blue indicates hazard impression below the median value, and red is above the median. SCC, Spearman’s correlation coefficient; FDR, false discovery rate.

The Spearman’s correlation coefficient (SCC) was used to identify consistent relationships between genes and DEGAS risk scores. These values were calculated for each sample individually, providing four values, one for each of the four samples. The SCC distributions varied among these samples (**Figure 4**A). Plotting the maximum and minimum SCC highlighted microseminoprotein-beta (*MSMB*) as the sole gene exhibiting a strong and consistent negative correlation with high-risk areas in all four samples (SCC range = [−0.339, −0.621], *P* value range = [< 1E−100, 7.98E−68]; Figure 4B). Conversely, many genes, like eukaryotic translation elongation factor 2 (*EEF2*), showed a consistent positive correlation with high-risk areas (SCC range = [0.288, 0.569], *P* value range = [2.96E−82, 3.64E−293]. Others demonstrated negative and positive correlations across different samples, like kallikrein related peptidase 3 (*KLK3*) (*i*.*e*., PSA gene, Normal = −0.303, Stage II = −0.594, Stage III = 0.562, Stage IV = 0.450), neuropeptide Y (*NPY*) (Normal = −0.295, Stage II = −0.422, Stage III = 0.601, Stage IV = 0.449), and *ubiquitin C* (*UCB*) (Normal = 0.587, Stage II = 0.666, Stage III = −0.328, Stage IV = 0.457, Figure 4C). Representative genes from each pattern are depicted in Figure 4D. Additionally, there is a relationship between DEGAS risk and total RNA expression in the normal tissue (SCC = 0.546, *P* = 1.15E−197), emphasizing that *MSMB* loss is counter to overall higher transcriptional activity in high-risk normal glands (Figure S6).

Both *MSMB* and *KLK3* serve as epithelial markers in prostate cancer [24]; however, their expression patterns diverged: *MSMB* expression was reduced in the high-risk benign-appearing glands (log_2_ fold change = −1.38, *P* = 2.05E−67) and consistently less expressed in tumor tissue. *KLK3* was expressed in both tumor and normal glands but was specifically downregulated in the highest-risk glands (log_2_ fold change = −1.16, *P* = 2.05E−67). *EEF2*, a translation elongation factor, has been implicated in promoting tumor progression across multiple malignancies, including prostate cancer. Its overexpression has been linked to increased protein synthesis, supporting cellular proliferation and metastatic potential [25]. *NPY*, a neuropeptide widely studied in stress responses, has been shown to influence tumor progression and the tumor microenvironment in prostate cancer, particularly through its interactions with the sympathetic nervous system and immune modulation [26]. UBC, encoding polyubiquitin, is a crucial component of the ubiquitin-proteasome system, regulating protein degradation and cellular stress responses. Dysregulation of ubiquitin pathways has been linked to oncogenesis and may contribute to tumor adaptation and progression [27].

Though these other genes have known roles in cancer, *MSMB* had the strongest correlation with DEGAS risk scores and was consistently negatively correlated with risk across all four samples. Moreover, it was significantly downregulated in the high-risk glands of the normal sample. Thus, we thought *MSMB* may serve as a specific marker for high-risk normal glands and be useful for patient risk stratification. Rather than immediately proceeding with proteomic validation, these results prompted us to first validate the high-risk gland-low *MSMB* relationship with an additional ST data set and investigate the immunological milieu using single-cell deconvolution methods.

### Validation cohort analysis

#### DEGAS identifies high-risk glands with loss of MSMB that coincides with increased myeloid-origin inflammatory cells and decreased T-cells and fibroblasts

**Figure 5:**
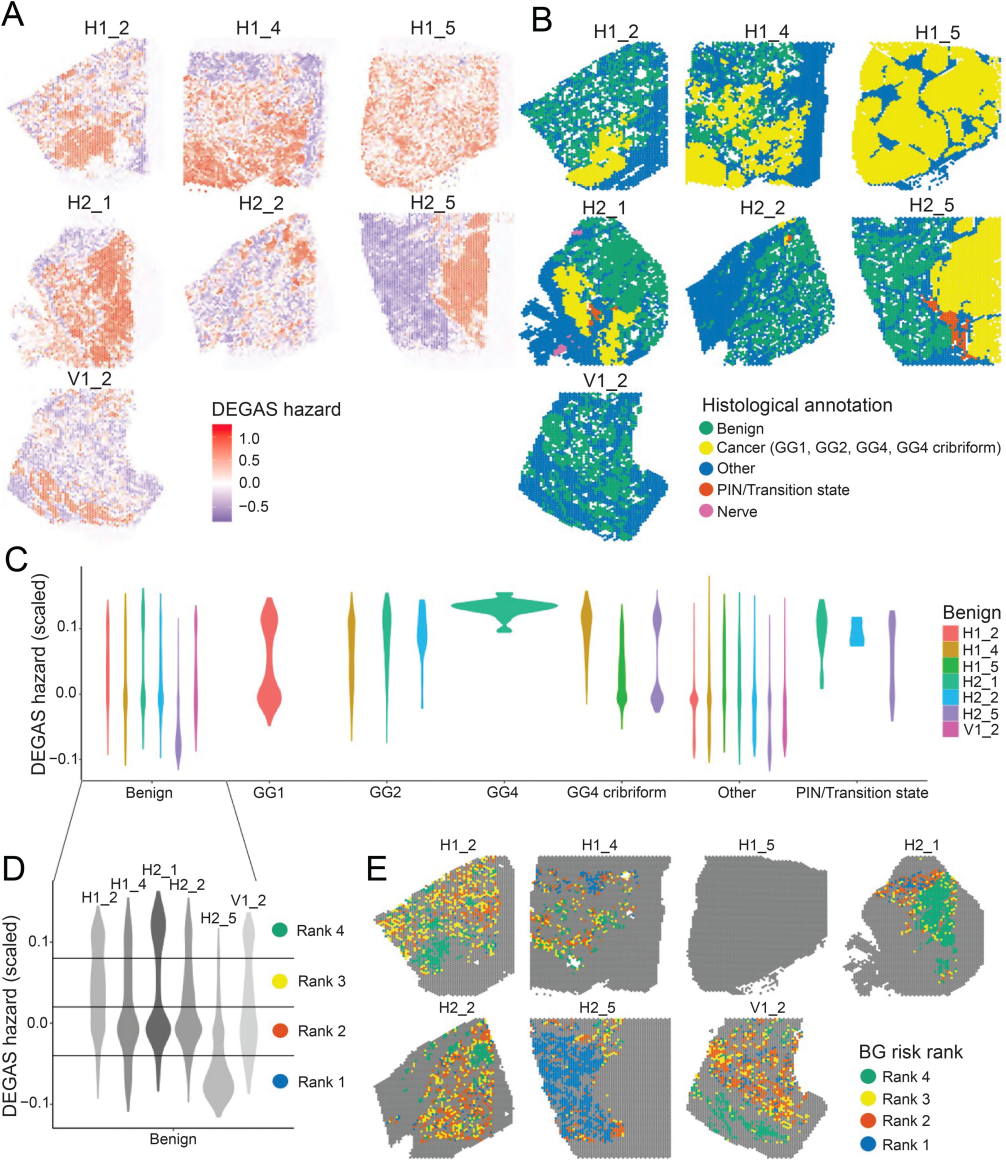
DEGAS highlights high-risk morphologically BGs with discrete spatial localization in external dataset **A**. DEGAS hazard impressions for seven Visium ST samples from the prostate of a single patient. **B**. Histology of both cancerous and benign across the seven samples. **C**. Violin plots of DEGAS hazards showing a trend between increasing tumor grade and hazard predictions across GG1 through GG4 regions. **D**. The morphologically benign tissues with different DEGAS Hazard distributions. They were divided into four ranks of increasing hazard for analysis. **E**. Visualization of the four ranks in the original histopathology. GG, Gleason grade.

To identify consistent and reproducible genetic findings, an additional independent prostate cancer ST dataset from Erickson et al. [28] was analyzed. This research group inferred DNA-copy number variations from ST, identifying that tumor tissue was clonally related to BGs. DEGAS also again highlighted regions of high-risk normal glands (**Figure 5**A and B). Some of the highest-risk regions (Figure 5A) were identified in a sample that comes from the opposite side of the prostate from the main cancer focus (Figure 5A). DEGAS risk predictions varied both within and between histologic tissues, emphasizing that histological labels alone are insufficient to identify tissue heterogeneity associated with prostate cancer progression (Figure 5C). The benign morphology glands had large variations in risk scores (Figure 5D). To create categorical variables for visualization, the BGs were separated into four groups of increasing risk at roughly equally spaced DEGAS risk score intervals (Figure 5D). There were 1655 spots in rank 1, 1039 spots in rank 2, 1402 spots in rank 3, and 882 spots in rank 4. BG rank 1 (lowest risk) rarely overlapped with BG rank 4 (highest risk), whereas BG ranks 2 and 3 have intermediate risk and show more spatial colocalization (Figure 5E).

**Figure 6:**
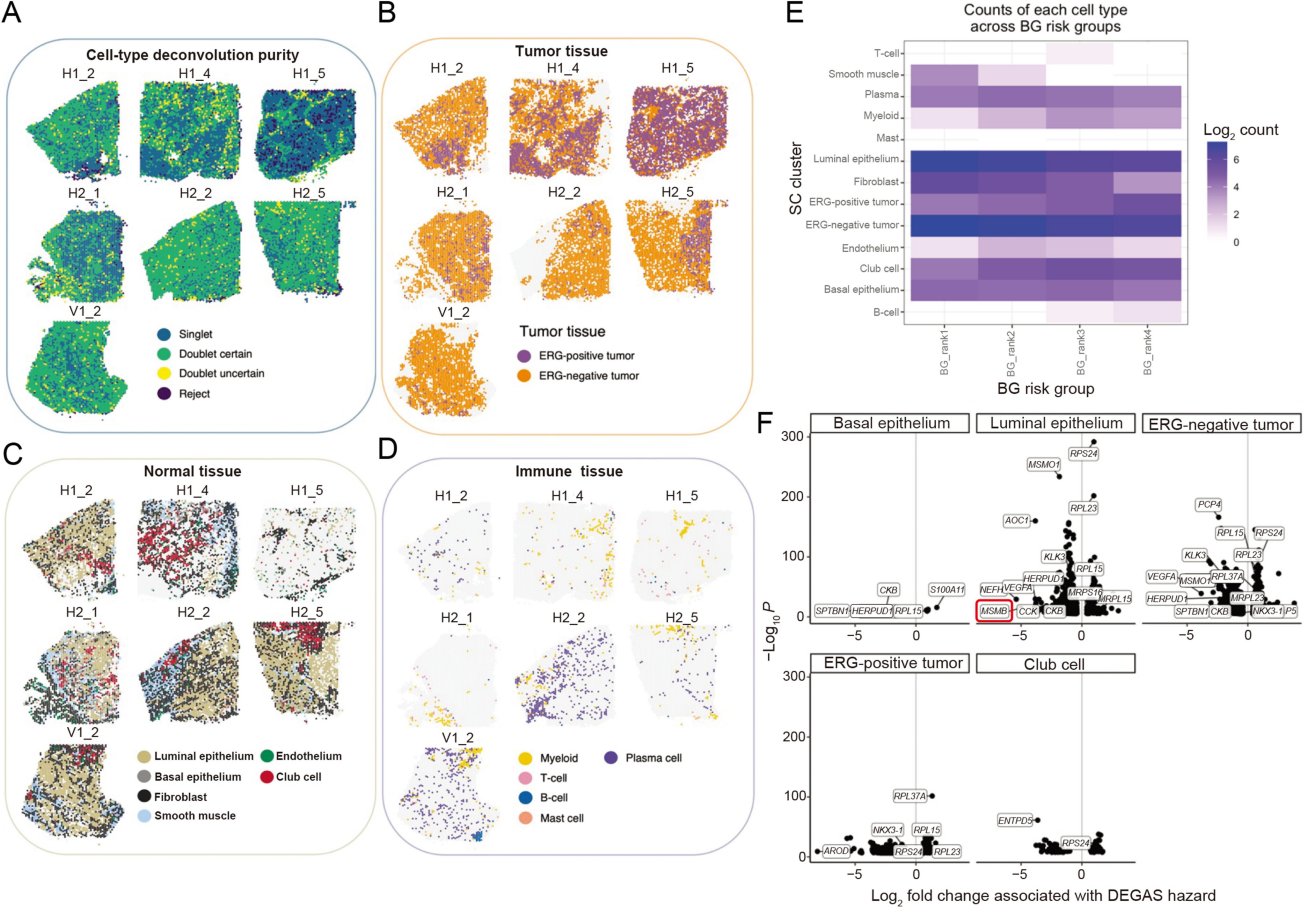
ST-spot deconvolution with annotated single-cell dataset and cell-specific differential expression associated with DEGAS risk **A**. Cell deconvolution/decomposition at each ST spot by the RCTD attributes SCs to each ST spot. If there are multiple predominant cell types at a location, RCTD provides “doublets”. Certain locations cannot be confidently decomposed and are called “reject”. H1_5 is the majority cancer sample and is attributed mostly to a SC type. Other samples show greater heterogeneity in the number of cell types ascribed to each location. **B**. Both ERG-positive and negative tumor cells are mapped in both tumor and non-tumor histological regions. **C**. Normal tissue contains predominantly luminal epithelia, and tumors also have luminal cells. **D**. Immune cells, mostly myeloid and plasma cells, are scattered throughout each sample. There is a group of B-cells in the lower right corner of the V1_2 sample. **E**. Cell-type composition of the four morphologically BG risk shows increasing populations of myeloid cells, B-cells, and fewer luminal endothelia, fibroblasts, and smooth muscle. **F**. Cell-type specific differential expression associated with DEGAS risk scores shows *MSMB* downregulation is specific to luminal epithelia (red rectangle).

Because each ST spot contains multiple cells, the Robust Cell Type Decomposition (RCTD) cell type deconvolution algorithm was employed to discern how DEGAS risk relates to cell-type abundance. Using an annotated prostate cancer SC dataset from Song et al. [29], RCTD was able to confidently assign SC clusters to most regions of the ST data (**Figure 6**A). Erythroblast transformation-specific related gene (ERG)-positive tumor cells from the SC were associated with the tumor-dense areas of the ST samples (Figure 6B). Deconvolution of the benign regions revealed a mix of both ERG-negative tumor cells and normal epithelial cells (Figure 6C), and a diversity of immune cell types in both tumor and normal tissue (Figure 6D).

Cell-type composition varied across the four different risk groups (Figure 6E). The Spearman’s correlations between DEGAS risk scores and RCTD cell-type enrichment among the histologically normal glands were calculated (Figure S7). The three strongest positive correlations were B-cells (SCC = 0.25, *P* = 1.15E−72), plasma cells (SCC = 0.25, *P* = 1.15E−72), and myeloid cells (SCC = 0.19, *P* = 5.41E−42). The three strongest negative correlations were fibroblasts (SCC = −0.27, *P* = 5.90E−82), T-cells (SCC = −0.16, *P* = 3.91E−25), and basal epithelia (BE, SCC = −0.15, *P* = 1.44E−27). We note that ERG-positive epithelial cells increased (SCC = 0.16, *P* = 6.55E−30) and ERG-negative epithelial cells decreased (SCC = −0.14, *P* = 2E−22). The tool Cell Type-Specific Inference of Differential Expression (C-SIDE) was further used to understand how gene expression of each cell type changed with DEGAS risk, while controlling for these changing cell type proportions. This analysis showed that *MSMB* downregulation is specific to high-risk luminal epithelial cells, recapitulating the results of the discovery set (Figure 6F).

#### Deep learning image analysis reveals subtle similarities between high-risk glands, high-grade cancer, related to increasing inflammation, features of high-grade cancer, and descreasing stroma

**Figure 7:**
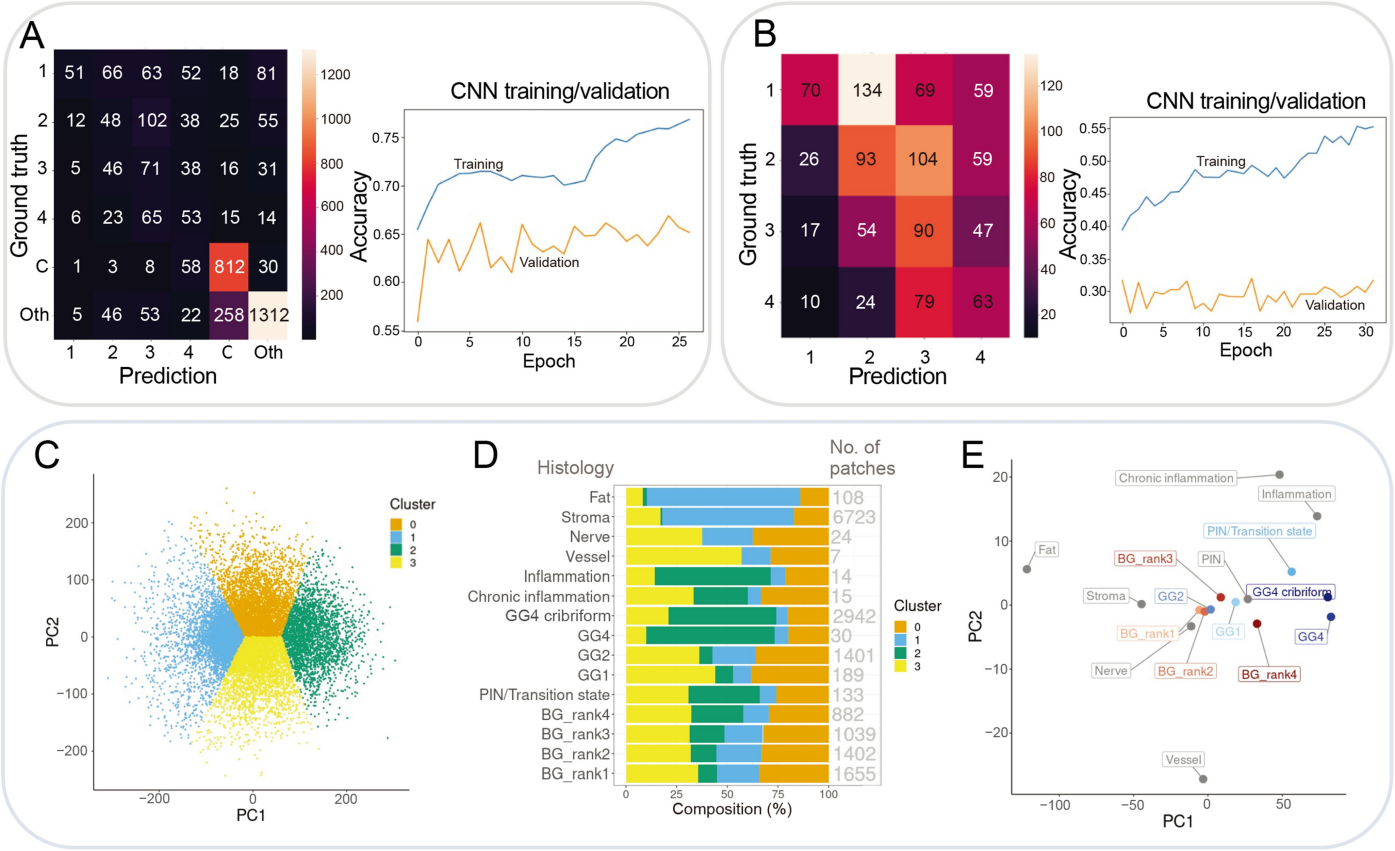
Clustering of image patches reveals subtle similarities between high-risk prostate glands and high-grade cancer **A**. Classification of sample groups by confusion matrix and training log of EfficientNetB4 CNN fine-tuned. Six groups were classified: The four BG risk groups, cancer, and all other histology grouped under the label “other”. The CNN accurately identified only the cancer and “other” regions (“C” and “Oth”). **B**. Classification of the four BG risk groups by a separately fine-tuned CNN. Due to the heavy class imbalance, a separate CNN was fine-tuned to classify only the four BG risk groups. CNN performance in the validation data is not better than random classification and shows evidence of overfitting. **C**. K-means clustering of patches based on two principal components from PCA of autoencoder embeddings. PCA of autoencoder embeddings for patches from all samples. Color represents K-means clustering based on two principal components. **D**. Proportion of clusters across histology. The proportion of these clusters in different histology shows that Cluster 2 (green) increases in increasingly high-risk normal glands. Cluster 2 is strongly represented in the Chronic inflammation, Inflammation, PIN/Transition state, and high-grade cancer tissue (GG4, GG4 cribriform). Cluster 2 is largely absent from the vessel, stroma, nerve, and fat regions. Cluster 1 is highly represented in Fat, stroma, nerve, and vessel, decreasing in proportion with increasing BG risk rank. **E**. Calculation and plotting of the mean PCA embedding. Fat, stroma, nerve, and vessels were separated from epithelial and inflammatory histology. Centroids of BG risk ranks 1 through 4 progresses sequentially in the direction of GG4 tumor, aligning with increasing DEGAS risk scores. CNN, convolutional neural network; PCA, principal component analysis; PC, principal component; val, validation.

Deep learning image analysis was used to determine whether there were subtle morphological differences in prostate tissue histology that coincided with these transcriptomic changes. The ability to recognize high-risk glands with myeloid infiltration would be a useful clinical and research tool, so a state-of-the-art convolutional neural network (CNN) was fine-tuned to classify the ST data into six categories: the four BG risk groups, cancer tissue, and a collective “other” category for all remaining histology. This CNN displayed high accuracy in identifying cancer and “other” histology but failed to differentiate the BG risk groups (**Figure 7**A). There were significantly more “cancer” and “other” patches, which may have biased the model, so a CNN was trained to differentiate just the four BG risk groups. This model was only capable of near-random accuracy and showed overfitting to the dataset. This highlights the need for multi-omics, and specifically ST, to capture both the molecular and morphological profiles underlying the field effect (Figure 7B).

Taking an unsupervised deep learning approach, an autoencoder was used to compress the histological images. Principal component analysis (PCA) of the compressed images revealed a point cloud, and K-means was used to divide this point cloud (Figure 7C). PC1 and PC2 represented 10.8% and 4.6% of the variance of the ∼ 200,000 image features. The proportion of these four clusters was compared to histological labels, which provided interpretability (Figure 7D): Cluster 2 was strongly represented in the highest-grade tumor tissue (GG4, GG4 cribriform), chronic and acute inflammation, and intraepithelial neoplasia. Cluster 1 was strongly represented in fat and stromal tissue. Clusters 0 and 2 did not show an obvious pattern of enrichment that aligned with a histology. Crucially, as the DEGAS risk score of normal-appearing glands increased, these regions had increased proportions of Cluster 2 and decreased proportions of Cluster 1 (*P* = 3.85E−28), reflecting an increase in inflammation-cancer image signature and a decrease in normal fat-stromal image signature. The standardized Chi-squared residual for Clusters 2 and 1 in the BG rank 4 glands were the largest significant residuals: 10.11 and −5.62, respectively.

When a PCA centroid was calculated for each histological label, the clusters were clearly separable (Figure 7E). Close inspection revealed a parallel progression of BG rank 1 to BG rank 4 glands, as well as benign stromal tissues towards inflammatory tissue and high-grade Gleason grade 4 (GG4) cancer along the first principal component. These image results align directly with the DEGAS-derived transcriptomic findings, *i.e.*, high-risk morphologically BGs exist with an increased inflammatory and decreased stromal components. This trend was also identified when correlating the mean loading of the first 30 components (48% of variance in data) with the DEGAS risk scores (Figure S8).

### IHC Validation

#### Protein expression of MSMB is highly associated with 5-year distant metastasis, with normal tissue having more predictive power than tumor regions

To validate our spatial transcriptomic findings at the protein level, we performed IHC staining for MSMB (PSP-94) in FFPE prostatectomy samples from patients who either developed distant metastases within five years (*n* = 4) or remained metastasis-free for at least five years (*n* = 4). All patients had International Society of Urological Pathology (ISUP) Grade 5 primary tumors, and three of four patients in each group had positive lymph nodes at diagnosis. Age across the two groups was not significantly different (two-tailed Wilcoxon *P* = 0.4651, Table S2). These well-balanced baseline characteristics ensured that differences in IHC staining were not driven simply by tumor grade or stage.

**Figure 8:**
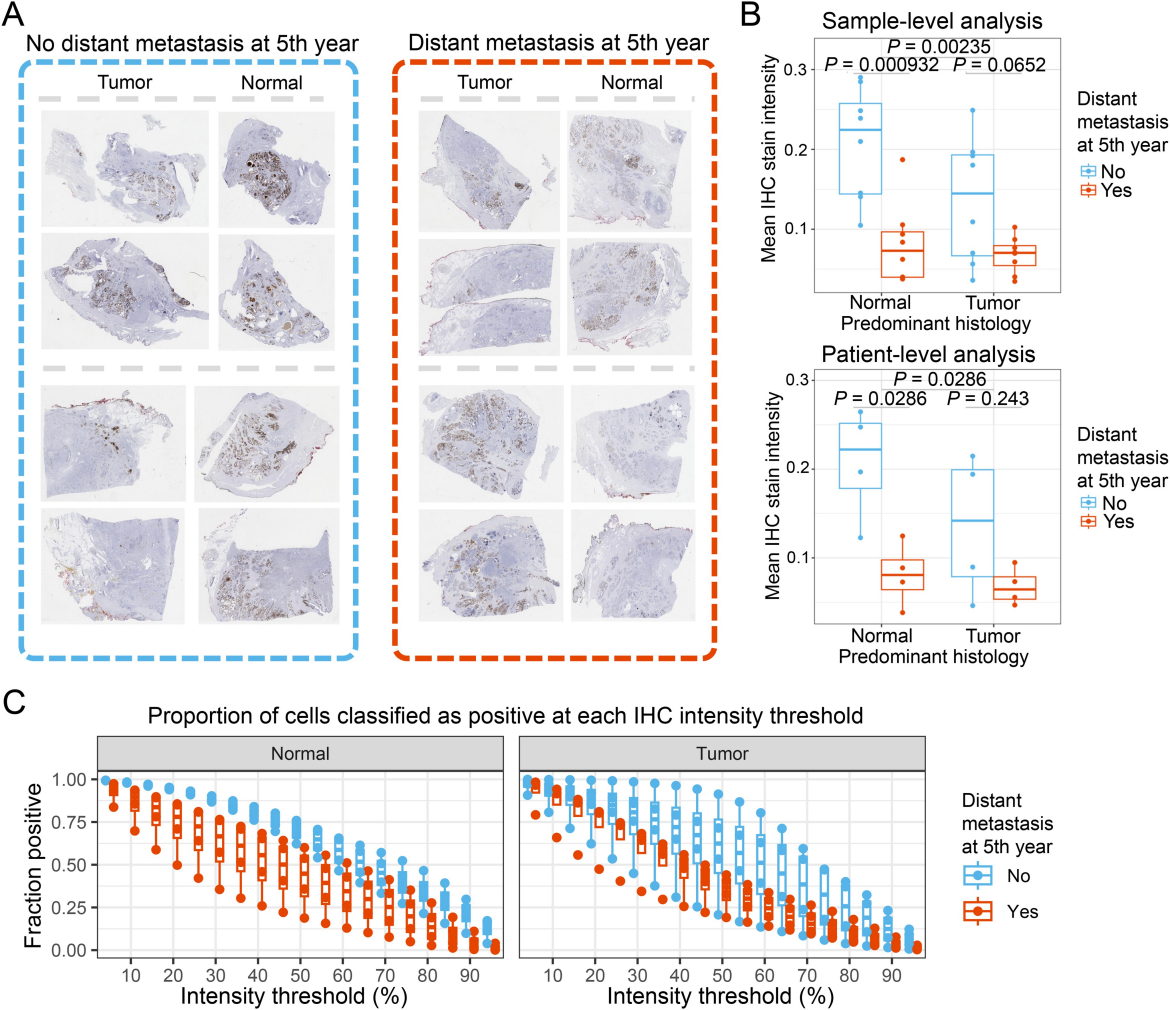
Loss of MSMB protein expression in BGs predicts 5-year distant metastasis in prostate cancer patients **A**. Representative IHC staining for MSMB protein in tumor and benign (normal) prostate tissue from patients with (red, right) and without (blue, left) 5-year distant metastases (40× magnification). Patients who developed distant metastases exhibited lower MSMB expression, particularly in BGs. **B**. Quantification of MSMB staining intensity at the sample level (top) and patient level (bottom). At the sample level, MSMB expression in normal glands was significantly lower in patients with distant metastases (*P* < 0.001), while tumor regions showed a weaker association (*P* = 0.005). At the patient level, MSMB staining in benign regions remained predictive of metastasis (*P* = 0.029), whereas tumor regions did not (*P* = 0.243). **C**. Threshold-based analysis of IHC staining intensity, showing the fraction of cells classified as MSMB-positive at increasing intensity thresholds. MSMB expression in normal prostate tissue consistently stratified metastatic and non-metastatic patients across a wide range of intensity thresholds, with a stronger effect observed in benign regions than in tumor regions. IHC, immunohistochemical.

Representative IHC images and quantification are displayed in **Figure 8**. The whole-slide image (WSI) thumbnails from four representative patients revealed a clear reduction in MSMB staining within normal tissue among those with distant metastasis (Figure 8A). Using QuPath to quantify the cytoplasmic IHC staining intensity for every cell within the parenchyma (non-stromal tissue) showed a significant ability to stratify patient prognosis, whether treating the images as independent samples (one-tailed Wilcoxon *P* = 0.00235), or analyzing the IHC staining intensity summarized to the patient level (one-tailed Wilcoxon *P* = 0.0286, Figure 8B). Importantly, the difference in prognostic significance of the normal tissue was greater than that of tumor tissue at the patient level (one-tailed Wilcoxon *P* = 0.0286 *vs*. *P* = 0.243).

IHC intensity scoring in pathology is typically done by using an intensity threshold to define positive cells, and the fraction of positive cells is correlated to clinical outcomes [30,31]. The selection of the threshold is extremely important and susceptible to bias [32], so we systematically evaluated 20 different percentiles along the entire stain intensity distribution. The prognostic power of MSMB protein IHC remained robust across a wide range of intensity thresholds (Figure S9). In normal tissue, the fraction of cells classified as MSMB-positive, as defined by staining greater than the 50th percentile, clearly distinguished the metastatic from the non-metastatic group (AUC = 1, one-Tailed Wilcoxon *P* = 0.014). In contrast, tumor regions showed slightly weaker discriminatory power (AUC = 0.812, One-tailed Wilcoxon *P* = 0.1).

These results strongly support the notion that MSMB loss in ostensibly normal prostate glands, both at the transcriptomic and proteomic level, is linked to aggressive disease. Furthermore, the observation that normal tissue exhibited superior predictive capacity relative to tumor tissue underscores the relevance of a “field effect,” wherein molecular alterations in ostensibly benign regions may foreshadow metastatic progression. This protein-level validation of our transcriptomic findings suggests that measuring MSMB expression in BGs could serve as a promising biomarker strategy for identifying patients at higher risk of distant recurrence.

## Discussion

The goal of this study was to understand how different components of the prostate tumor, microenvironment, and ostensibly normal tissue in the same organ are associated with cancer progression. Utilizing over 400 RNA-seq samples, 11 ST samples across two independent datasets, single-cell data, and histopathological imaging, DEGAS repeatedly demonstrated that certain morphologically BGs are highly associated with prostate cancer progression. These high-risk glands exhibit upregulated RNA signatures of antigen presentation and neoplasms and are associated with the loss of *MSMB*. These molecular changes in benign-appearing glands suggest a role in cancer progression, and our future work will focus on confirming their contribution to carcinogenesis and distant spread. Our results build on RNA-seq research that has shown the prognostic value of normal-adjacent tissue in eight different cancers [33,34]. The growing availability of ST technologies will facilitate the continued high-resolution dissection of the microenvironment to understand how the complex interplay contributes to patient prognosis.

Integrating SC and ST by cell-type decomposition demonstrated that *MSMB* loss occurs specifically in luminal epithelia, the putative progenitor of prostate adenocarcinoma. Additionally, the decrease of *MSMB* coincides with infiltration by inflammatory cells of the myeloid lineage and reduction of fibroblasts and T-cells. Validating the transcriptomic results by analyzing ST histopathology demonstrated that distinguishing high and low-risk glands is difficult, but high-risk glands share subtle imaging similarities to high-grade cancer tissue associated with increased inflammation. Our findings support a hypothesis that the observed increases in antigen presentation signatures, myeloid inflammation, and loss of epithelial differentiation markers in benign-appearing glands may play a role in the link between chronic inflammation, immune desensitization, and the progression of prostate cancer.

Supporting our identification of field effects in high-resolution transcriptomics, there is substantial literature in pathology and molecular epidemiology linking *MSMB* [35,36], differentiation [24,37], chronic inflammation [38,39], and myeloid cells [40,41] to prostate cancer progression. Therefore, our future work will focus on leveraging these different components of the field effect as metastasis biomarkers. This has been preliminarily explored, and MSMB loss in normal glands was moderately predictive of higher prostate cancer grade and invasion outside prostate [42]. These experiments, however, relied on expert pathologists’ qualitative scoring and did not directly address metastasis. Metastasis prediction is the most important clinical need in prostate cancer management. Therefore, we are undertaking retrospective clinical validation of MSMB staining in prostatectomies from a case-control cohort of metastatic and non-metastatic patients. Additionally, the use of digital image analysis allows for direct quantification of MSMB staining, potentially improving metastasis prediction.

We hope in our future studies to address the technical limitations of the currently available assays and methodologies enumerated here. Visium ST provides a snapshot in time, limiting the ability to discern dynamic processes in the microenvironment. Therefore, it cannot be directly discerned whether these high-risk glands become malignancies or whether they contribute to the progression of existing malignancies. This conundrum is especially interesting because almost all prostate cancers are polyclonal: sectioning the entire prostate will reveal several simultaneous tumors of different genetic origin [9]. Intriguingly, it has been shown that some prostate cancer metastatic foci are derived from lower-grade progenitor cancer cells, rather than the bulk, higher-grade tumors that brought on clinical suspicion [43]. This is counter to traditional pathology and bioinformatics thought. MSMB and myeloid cells have not been studied simultaneously, and their spatial colocalization may be of key importance to prognosis. Broader validation of the prognostic value of MSMB and myeloid inflammation at the proteomic and transcriptomic level is needed, especially in cohorts of diverse racial and ethnic backgrounds for whom there are known differences in outcomes [44] and tumor biology [45,46].

Using our deep transfer learning methods, we identified morphologically BGs that exhibited loss of differentiation markers and increased signatures for antigen presentation and aggressive disease. However, further studies are needed to confirm whether these changes contribute directly to cancer progression. These same high-risk morphologically BGs showed increased myeloid infiltration, which was only revealed via SC integration techniques. Fine-tuned deep learning image analysis revealed subtle changes in morphology related to inflammation. Markers for these high-risk, morphologically BGs, in conjunction with PSA and other demographic information, hold potential as part of the next generation of precision prostate oncology testing, pending further validation.

Our IHC validation of MSMB protein loss in normal prostate glands supports these findings and underscores the importance of the “field effect”. Notably, we observed that MSMB expression had greater prognostic utility in benign tissue than in tumor regions, suggesting that transcriptional and proteomic alterations in ostensibly normal glands may precede or drive aggressive behavior. This is consistent with prior studies linking chronic inflammation and subtle epithelial changes to malignant progression.

Importantly, the strength of this association is particularly striking given that our cohort consisted of just 8 patients with the same ISUP Grade 5 tumors, many of whom also had positive lymph nodes at diagnosis. These patients, under current clinical guidelines, would all be classified as high-risk and managed similarly. The fact that MSMB expression in morphologically BGs could perfectly distinguish which patients developed distant metastases — despite identical clinical staging — suggests that it captures an aspect of disease biology not currently incorporated into standard prognostic models. This highlights MSMB as a compelling biomarker candidate for refining metastatic risk stratification, and the low-expression tissue regions as important foci for targeted omics analyses.

Future studies will focus on combining MSMB expression in BGs with other established clinical markers (*e.g.*, PSA, Gleason grade, and genomic tests) to further refine risk stratification for metastatic progression. Such multimodal biomarker panels could be particularly valuable for identifying men who would benefit from intensified surveillance or adjuvant therapies. Ultimately, integrating ST, single-cell analysis, and advanced imaging-based pathology scoring may provide a comprehensive toolkit for precision oncology in prostate cancer.

## Materials and methods

### DEGAS framework with new applications on spatial transcriptomic data

The deep transfer learning framework DEGAS [16] is constructed using TensorFlow in Python [47] and wrapped with an R interface for broader accessibility [48]. In essence, this algorithm employs maximum mean discrepancy loss to synchronize the distributions of the two different datasets within a latent space. This facilitates the transfer of labels and patient outcomes (*e.g.*, mutational status and survival) between the datasets. This algorithm’s design, specifically the shared gene-level input features between ST and SC data, enables its effective application to ST data [49]. Applying DEGAS to ST data is particularly critical as it bridges gene expression and histopathology, offering a powerful tool to identify prognostic regions in the tumor and microenvironment. In this work, we trained DEGAS to predict “progression-free survival” using bulk patient tissue and mapped this information onto the ST. DEGAS can be downloaded from GitHub at https://github.com/tsteelejohnson91/DEGAS.

### Data pre-processing, feature selection, and analysis with DEGAS

All gene expression data (Table 1) were processed with DEGAS’s *preprocessCounts* function, which performs a log_2_ transformation and Z-score normalization, then scales RNA counts to a [0, 1] range. DEGAS was used in the standard DenseNet configuration with three hidden layers and five-fold bootstrap aggregation. Risk scores for ST data were normalized against the median, assigning “high-risk” to scores above zero and “low-risk” to those below. When handling multiple prostate samples, the DEGAS model was trained on a combined matrix, allowing for consistent risk score comparison across samples.

For feature selection, genes common to the ST and bulk tissue RNA-seq datasets were identified. Among these, the top 200 genes with the highest variability among The Cancer Genome Atlas (TCGA) patients served as input features for subsequent DEGAS models. To improve the generalizability of the models, ensemble learning principles were employed [50], aggregating training results from incrementally increasing feature sets, starting with 10 genes and doubling sequentially up to 200 genes to regularize the DEGAS risk predictions.

### Discovery cohort analysis

#### Differential gene expression and correlation of DEGAS risk score with gene expression

The DEGAS risk scores across the Normal, Stage II, Stage III, and Stage IV samples were compared using the Wilcoxon rank-sum test from the R *stats* package [48]. Differentially expressed genes (DEGs) between high and low-risk regions within the discovery dataset were also identified using the Wilcoxon test. The specific tissue regions under comparison for differential expression are detailed within the respective experiments. This non-parametric test statistic was chosen because it minimizes false positives without unduly sacrificing true positives [51]. *P* values were adjusted for the false discovery rate (FDR) utilizing the Benjamini-Hochberg (BH) method in the R package *stats* (v4·1·1) [52]. Volcano plots were generated using the log_2_ fold change. Significance was defined as adjusted *P* values below 1E−2. There was no significance cut-off fold-change.

Spearman’s correlation between DEGAS risk scores and gene expression was calculated for each ST prostate using the *cor* function from the R *stats* package. This metric was used to easily identify genes that were consistently correlated with risk scores across all samples. The Spearman’s correlation between genes and DEGAS risk scores was calculated in the discovery set, using all ST spots from all four samples, with no filtering by tissue type. Plotting the minimum and maximum of correlation coefficients among the four samples allowed for the identification of genes that were either consistently negative or positively correlated across all samples (*e.g.*, negative correlation in all samples, positive correlation in all samples). All *P* values were adjusted using the FDR-BH method.

#### Functional enrichment analysis of genes

Functional enrichment analysis was performed on the most DEGs within the discovery dataset. Up to 100 genes with FDR-adjusted *P* values smaller than 1E−3 and finite log_2_ fold change were input to Toppgene [53]. The *P* values reported by Toppgene were also adjusted using the Benjamini-Hochberg FDR method. Enrichment results with high similarity and near-identical gene sets were consolidated to maintain clarity.

### Validation cohort analysis

#### Integrating ST and SC data to identify cell-type specific differential expression in luminal epithelial cells

The RCTD tool [54] was employed to integrate SC and ST data (Table 1). RCTD decomposes the expression of each ST spot as a linear combination of pre-defined cell types from the reference SC dataset. This tool was applied to the seven ST samples in the validation set. Building on the RCTD results, C-SIDE [55] was then used to identify cell type-specific differential expression associated with DEGAS risk scores. All tools were used with their default settings. RCTD, specifically, was run in “doublet mode”, assigning up to two cell types to each ST spot. The “full mode” results from RCTD were used to calculate Spearman’s correlations between DEGAS risk scores in normal glands and the enrichment scores for each cell type at each ST spot. All *P* values were corrected using the FDR-BH multiple testing adjustment.

To explore cell type proportion changes in normal glands with varying risk levels, it was necessary to divide these tissues into different risk groups for analysis. The range of DEGAS risk scores was divided into four equal intervals, and glands were named BG rank 1, BG rank 2, BG rank 3, and BG rank 4 in order of increasing risk. This categorization enabled comparison of cell type counts assigned by RCTD doublet mode across the groups.

#### Deep learning image analysis

ST data contains both histopathological images and transcriptomic data. The 20× magnification image can be divided into 148 × 148 pixel image patches that correspond to each RNA probe. These patches were used to train two deep learning architectures: a CNN and an autoencoder. Accurate classification of high-risk glands has potential clinical significance, offering a less expensive alternative to ST data for identifying high-risk tumor regions in histopathology.

The *EfficientNetB4* CNN architecture was used for the image classification task [56]. This is the state-of-the-art CNN model that was selected for its balance between computational efficiency and accuracy. Images were resized from 148 × 148 to 256 × 256 pixels, as required by this architecture. Training was started with 80 epochs and early stopping based on validation loss, with patients of three epochs to mitigate overfitting. To improve generalizability, in-model data augmentation was achieved using the built-in Keras functions *RandomRotation, RandomTranslation, RandomFlip,* and* RandomContrast* sequentially. Initially, *EfficientNetB4* weights were locked to ImageNet’s pre-trained values [57], with training confined to the top layers. After this first phase of training, selective unfreezing of the top 20 layers — excluding *BatchNorm* layers — was performed to refine the model. The learning rate was reduced to 1E−4 to allow for slower adjustments during this fine-tuning stage.

An autoencoder was trained to compress patches from all histological regions. These compressed image representations were used to identify subtle similarities across the images. The AE autoencoder architecture was based on Hou et al. [58] and implemented in Keras [59]. The encoding layers were equipped with 100, 120, 240, and 320 kernels of varying sizes, followed by *BatchNorm* and *LeakyReLU* activations, with *AveragePooling* applied after every two convolutional layers. The corresponding decoder reconstructs the image by inversely mirroring the encoder structure. The model employed mean squared error reconstruction loss and an Adam optimizer [60]. Training was conducted for 300 epochs, incorporating early stopping with a patience of 3 epochs, a batch size of 16, and 10% of the patches reserved for validation. The autoencoder’s bottleneck layer provides a 200,000-feature representation of each image.

The dimension of these extremely large vectors was reduced using PCA in Python *scikit-learn* (v1.1.1) [61], and two components were retained for K-means clustering and visualization. PCA was used instead of non-linear dimensionality reduction techniques like UMAP and t-SNE because PCA better retains global data structure in low-dimensional embeddings. By retaining global structure of the data, distance in the low-dimensional space better reflects distance in the high dimensional space. Thus, if two image patches are nearby in PCA space, they might also be nearby in high dimensional space. This interpretability allowed the identification of subtle similarities in the complex histological images. Two components were retained for visualization and clustering. K-means clustering was used because of its ability to partition a point cloud into equal pieces. The objective was not to identify discrete clusters but instead to define broad categories of data. Given two PCA axes, K-means of four was selected to provide four groups like a confusion matrix (−PC1 & −PC2; +PC1& −PC2; −PC1 & +PC2; +PC1 & +PC2).

### IHC Validation

#### Protein-level quantification of MSMB expression and correlation with 5-year distant metastasis

Four patients were identified with ISUP Grade 5 prostate cancer who suffered distant metastasis to bone or another organ, and not solely regional lymph nodes or local recurrence. These four patients were matched to four patients with identical ISUP grade and N-stage, using a cancer registry at the Indiana University School of Medicine. The matched control patients did not suffer distant recurrence within five years, and all four matched control patients had more than seven years of follow-up, also without distant recurrence. The ages of patients identified in two outcome groups were compared to evaluate age-related biases.

ISUP grading involves combining the two highest Gleason grades of the tumor mass of the prostatectomy specimen. The diagnostic H&E-stained specimens were used to identify the tissue with the largest quantities of the primary and secondary grades. In addition, two sections of normal tissue were selected. One of the pieces of normal tissue was adjacent to the primary tumor mass, and an effort was made to select a piece of normal tissue that was contralateral to the side of the prostate containing the primary tumor mass, and thus, potentially the least affected by local neoplastic inflammation. Selection of all tissues was performed by our expert pathologist.

The protein product of the *MSMB* gene has several aliases, including “PSP-94”. Rabbit monoclonal antibodies for this protein were purchased from Abcam^®^ (EPR7346, Catalog No. ab133296) for IHC. These antibodies were validated with endometrium serving as a negative control and non-cancerous glandular prostate tissue as a positive control. The dilution of 1:5000 was the least dilute aliquot that had no staining of background stroma and retained strong staining of benign prostatic epithelium. The generated IHC slides of representative sections were digitized with a Leica Aperio CS2 scanner at 40× magnification. The digital WSIs were analyzed with the software QuPath, which enabled us to extract the mean cytoplasmic staining from all cells within the parenchymal tissue. The analysis of this image feature, called IHC intensity herein, is described in the relevant sections of the results.

## Supplementary Material

qzaf119_Supplementary_Data

## Code availability

Code for both DEGAS and deep-learning image analysis in this project is available within the Mendeley repository (https://data.mendeley.com/; DOI: 10.17632/97s9fjn5bb.1).

## CRediT author statement

**Justin L. Couetil:** Conceptualization, Data curation, Formal analysis, Investigation, Methodology, Software, Validation, Visualization, Writing – original draft, Writing – review & editing. **Ziyu Liu:** Formal analysis, Methodology, Software, Writing – review & editing. **Chao Chen:** Funding acquisition, Writing – review & editing. **Ahmed K. Alomari:** Funding acquisition, Resources, Supervision, Validation, Writing – review & editing. **Kun Huang:** Funding acquisition, Resources, Supervision, Writing – review & editing. **Jie Zhang:** Conceptualization, Funding acquisition, Resources, Supervision, Writing – review & editing. **Travis S. Johnson:** Conceptualization, Data curation, Funding acquisition, Project administration, Resources, Software, Supervision, Writing – review & editing. All authors have read and approved the final manuscript.
