# Supplementary material for: Deep Transfer Learning Links Benign Glands to Prostate Cancer Progression via Transcriptomics
Source: Genomics Proteomics Bioinformatics. 2025 Nov 29;23(6):qzaf119. doi: 10.1093/gpbjnl/qzaf119 (PMC13222491; doi:10.1093/gpbjnl/qzaf119)
Supplement: qzaf119_Supplementary_Data [file qzaf119_supplementary_data.zip › File S1.docx]

**File S1 Supplementary methods**

Preprocessed data and code for training DEGAS, autoencoders, and running PCA on the bottleneck embeddings are available on the Mendeley repository for this study (DOI: 10.17632/97s9fjn5bb.1). Vignettes for using DEGAS are available at <https://github.com/tsteelejohnson91/DEGAS>. The datasets analyzed during the current study are available in the 10x Genomics public repository (<https://www.10xgenomics.com/resources/datasets>). Erickson et al. data is available on Mendeley (<https://data.mendeley.com/datasets/svw96g68dv/4>). Song et al. data is available at GSE176031. TCGA-PRAD project data is available at the TCGA repository (<https://portal.gdc.cancer.gov/projects/TCGA-PRAD>). The preprocessed transcriptomic and imaging data used for the analysis presented herein are available at Mendeley (DOI: 10.17632/97s9fjn5bb.1).

Bulk RNA-seq and clinical data from prostate cancer patients were generated by Memorial Sloan Kettering as part of The Cancer Genome Atlas (TCGA-PRAD) project (Table 1) [1]. Published in 2018, this project used prospectively and retrospectively collected samples to chart the landscape of genetic and morphologic variations in 11 different human cancers [2]. This MSK cohort contains 493 prostate cancer patients with bulk RNA-sequencing of the primary lesions. The cohort contains patients of Stages I through IV, with most being Stage II and III. At baseline, 342 patients had no lymph node spread (localized disease), 78 patients had lymph spread (regional disease), and 73 had no information. This aligns with known prevalence of prostate cancer, where most patients have localized disease [3]. Tumor stages from T2A to T4 were represented in this cohort, with a majority of T2C, T3A, and T3B disease. Patients had a mean age of 61 years (std. 6.85). The outcome of interest was progression-free survival. Progression is defined by new tumor events, which can be distant metastasis, spread to regional lymph nodes, local recurrence, or death [4].

The prostate cancer discovery dataset (Table 1) comprises four 10x Genomics Spatial Transcriptomics (ST) prostate samples, including one from normal tissue and three from patients with Stage II, III, and IV prostate cancer [5]. All tissues were FFPE. These samples contained limited metadata. All biological samples were obtained from Indivumed Human Tissue Specimens. No other information for the normal prostate sample was provided. The Stage II and III, and IV samples all had total Gleason scores of 7. Grade represents the appearance of a tumor under the microscope. Higher grade tumors are less differentiated and correlate with a worse prognosis. Stage reflects the extent of tumor spread, increasing with direct extension outside the organ.

The validation dataset, comprised of seven ST samples from a single patient, was provided by the Lamb lab at the University of Oxford and the Lundeberg lab at KTH Royal Institute of Technology [6]. This study was mean to study copy-number variations in morphologically benign tissue, and how this defined clonal relationships between normal glands and tumors. This dataset therefore is directly relevant and appropriate for use in our study. Seven ST samples were obtained from the prostate of an 82-year-old patient. The initial tumor biopsy grade was Gleason 7 and updated to Gleason 8 (ISUP 4) upon prostatectomy. The seven samples are histologically diverse, including histologically normal glands both tumor-adjacent and on the opposite anatomical side of the prostate.

The SC data for our study was provided by the Huang lab at UCSF and Shalek lab at MIT [7]. This data was used to explore the transcriptional similarities between normal and malignant prostate epithelial cells, and therefore relevant to our work. These researchers obtained tumor biopsies from 11 patients with total Gleason scores ranging from 6 to 9. Four patients also provided matched normal tissue based on pathological assessment. These patients were largely treatment naïve, with only one patient on finasteride, providing a homogenous group of patients where transcriptomic differences are unlikely to be confounded by medical interventions. The researchers annotated SC clusters with *singleR* [8] for primary cell types. Due to a lack of comprehensive reference data, epithelial cell clusters were manually annotated. Their annotations encompassed Erg+ tumor, Erg- tumor, luminal epithelia, basal epithelia, and various other cell types. ERG is frequently translocated in prostate cancer [9]. One cell cluster, “myeloid cells” is comprised of macrophages, monocytes, and other myeloid cells.

**References**

[1] Broad institute tcga genome data analysis center. Broad Institute of MIT and Harvard. 2016.

[2] Sanchez-Vega F, Mina M, Armenia J, Chatila WK, Luna A, La KC, et al. Oncogenic signaling pathways in the cancer genome atlas. Cell 2018;173:321–37.e10.

[3] Institute NC. Seer*explorer: an interactive website for seer cancer statistics. 2023.

[4] Liu J, Lichtenberg T, Hoadley KA, Poisson LM, Lazar AJ, Cherniack AD, et al. An integrated tcga pan-cancer clinical data resource to drive high-quality survival outcome analytics. Cell 2018;173:400–16.e11.

[5] Human prostate 10x visium spatial gene expression dataset by space ranger 1.3.0. In: Genomics x, editor.

[6] Erickson A, He M, Berglund E, Marklund M, Mirzazadeh R, Schultz N, et al. Spatially resolved clonal copy number alterations in benign and malignant tissue. Nature 2022;608:360–7.

[7] Song H, Weinstein HNW, Allegakoen P, Wadsworth MH, Xie J, Yang H, et al. Single-cell analysis of human primary prostate cancer reveals the heterogeneity of tumor-associated epithelial cell states. Nature Communications 2022;13:141.

[8] Aran D, Looney AP, Liu L, Wu E, Fong V, Hsu A, et al. Reference-based analysis of lung single-cell sequencing reveals a transitional profibrotic macrophage. Nature Immunology 2019;20:163–72.

[9] Tomlins SA, Rhodes DR, Perner S, Dhanasekaran SM, Mehra R, Sun XW, et al. Recurrent fusion of tmprss2 and ets transcription factor genes in prostate cancer. Science 2005;310:644–8.
