# Supplementary material for: Deep Transfer Learning Links Benign Glands to Prostate Cancer Progression via Transcriptomics
Source: Genomics Proteomics Bioinformatics. 2025 Nov 29;23(6):qzaf119. doi: 10.1093/gpbjnl/qzaf119 (PMC13222491; doi:10.1093/gpbjnl/qzaf119)
Supplement: qzaf119_Supplementary_Data [file qzaf119_supplementary_data.zip › Supplementary material captions.docx]

**Supplementary materials**

**File S1 Supplementary methods**

**Figure S1 Independent feature selection for DEGAS analysis**

The 200 most variable genes intersecting the 10x data and TCGA show different variance from, and some are absent in, the Erickson et al. data. This highlights potential variations in biology or sample preparation. Thus, for each dataset, it is necessary to identify genes shared between bulk and ST data before identifying variable genes in the bulk data. TCGA, The Cancer Genome Atlas; ST, spatial transcriptomics.

**Figure S2 Effect of feature selection on hazard output**

Similar patterns emerge across different levels of feature selection. Taking the sum of predicted hazards across increasing feature sets provides a regularized DEGAS output. DEGAS, Diagnostics Evidence Gauge of Single cells.

**Figure S3 Normal tissue and Stage IV adenocarcinoma high risk regions**

**A.** Highest risk glandular region in the normal prostate tissue. There are no obvious signs of atypia. **B.** Highest risk cancer regions tend to have back-to-back glands or completely dissociated single cancer cells, aligning with higher Gleason scores. Regions of high-risk morphologies are visualized.

**Figure S4 Overlap in upregulated genes in high hazard regions across all four prostate ST samples**

Wilcoxon test was used to generate *P* values for differential expression of high and low hazard ST spots for each sample. Among the genes with BH FDR adjusted *P* values less than 1E−2, up to 100 genes were input to Toppgene for functional enrichment. The overlap between GO ontology categories of *Molecular Function, Biological Process, Cellular Component, Human Phenotype, Mouse Phenotype, Pathway, Interaction,* and *Disease* are visualized with this Venn diagram. ST, spatial transcriptomics; BH, Benjamini Hochberg; FDR, false discovery rate; GO, gene ontology.

**Figure S5 High Grade PIN genes from normal glands**

The gene module *High Grade Prostatic Intraepithelial Neoplasia* module is enriched in upregulated genes from the high-risk normal glands. The genes contributing to this module are *ACTB, CTNNB1, GSTP1, IGFBP3,* and *SQSTM1*. The expression of these genes is plotted on the four prostate discovery samples.

**Figure S6** **DEGAS hazard output correlates with total RNA expression and low-risk glandular regions express more *KLK3***

**A.** DEGAS-identified high-risk regions tend to align with higher levels of (**B**) total RNA expression. The Spearman correlation between these variables is 0.546. **C.** *KLK3* is the gene of the serum protein prostate serum-antigen and is expressed by prostatic glandular tissues (including cancerous tissue). Some of the highest-risk regions show no *KLK3* expression (blue circle), and the lowest-risk regions show high *KLK3* expression (green circle), despite being glandular tissue. **D.** K-means cluster (K = 2) show the regions of glandular (blue) and stromal prostate tissues (red). DEGAS, Diagnostic Evidence Gauge of Single cells.

**Figure S7 SCC of DEGAS risk scores and RTCD cell-type enrichment**

**A.** Spearman correlation between RCTD cell-type enrichment results and DEGAS risk scores among normal tissue ST regions, ordered by decreasing spearman correlation. Red line reflects locally weighted scatterplot smoothing. **B.** These same results are displayed in a volcano plot, showing fibroblasts have the strongest negative SCC with DEGAS risk scores. B-cells, Plasma cells, and Myeloid cells have the strongest positive SCC relationship. SCC, Spearman correlation coefficient; DEGAS, Diagnostic Evidence Gauge of Single cells; RCTD, Robust Cell Type Decomposition; ST, spatial transcriptomics; BE, basal epithelial; LE, luminal epithelial.

**Figure S8 Mean loading of first 30 principal components correlated with DEGAS risk scores**

Mean of loadings for the first thirty principal components is correlated with DEGAS risk scores. PC embeddings and DEGAS risk scores for BG ranks 1 through 4 are positively correlated. DEGAS, Diagnostic Evidence Gauge of Single cells; GG, Gleason grade; BG, Benign gland; PC, principal component.

**Figure S9 Threshold-Based Analysis of IHC Positivity and Its Association With 5-Year Distant Metastasis.**

**A.** At each IHC intensity threshold, the fraction of cells classified as positive was compared between samples with and without 5-year distant metastasis, stratified by histology (Normal *vs*. Tumor). Wilcoxon and T-tests were performed to quantify separation at the different intensity thresholds (**B** and **C**). **D.** ROC AUC for distinguishing patients with and without 5-year metastasis was calculated across the range of intensity thresholds. Normal tissue consistently and significant stratifies patient 5-year distant metastasis outcomes across the entire range of intensity thresholds. ROC, receiver operating curve; AUC, area under the curve.

**Table S1 Functional enrichment of upregulated genes in high hazard region**

**Table S2 Patient Baseline Clinical Data**
