# Supplementary figures and images for: Deep Transfer Learning Links Benign Glands to Prostate Cancer Progression via Transcriptomics
Source: Genomics Proteomics Bioinformatics. 2025 Nov 29;23(6):qzaf119. doi: 10.1093/gpbjnl/qzaf119 (PMC13222491; doi:10.1093/gpbjnl/qzaf119)

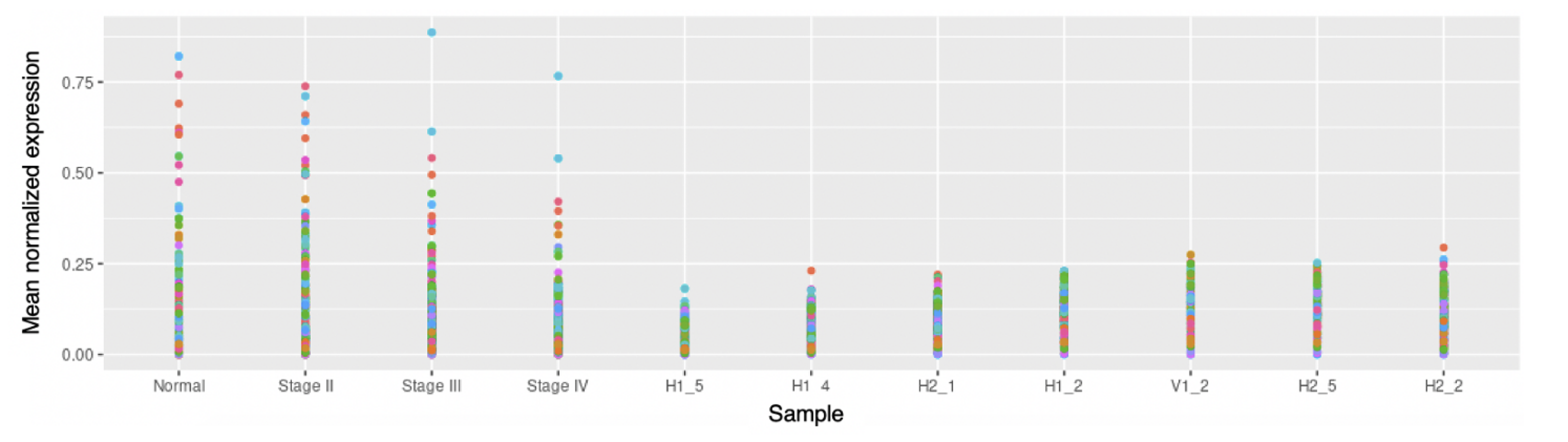

Supplement: qzaf119_Supplementary_Data [file qzaf119_supplementary_data.zip › Figure S1.tif]

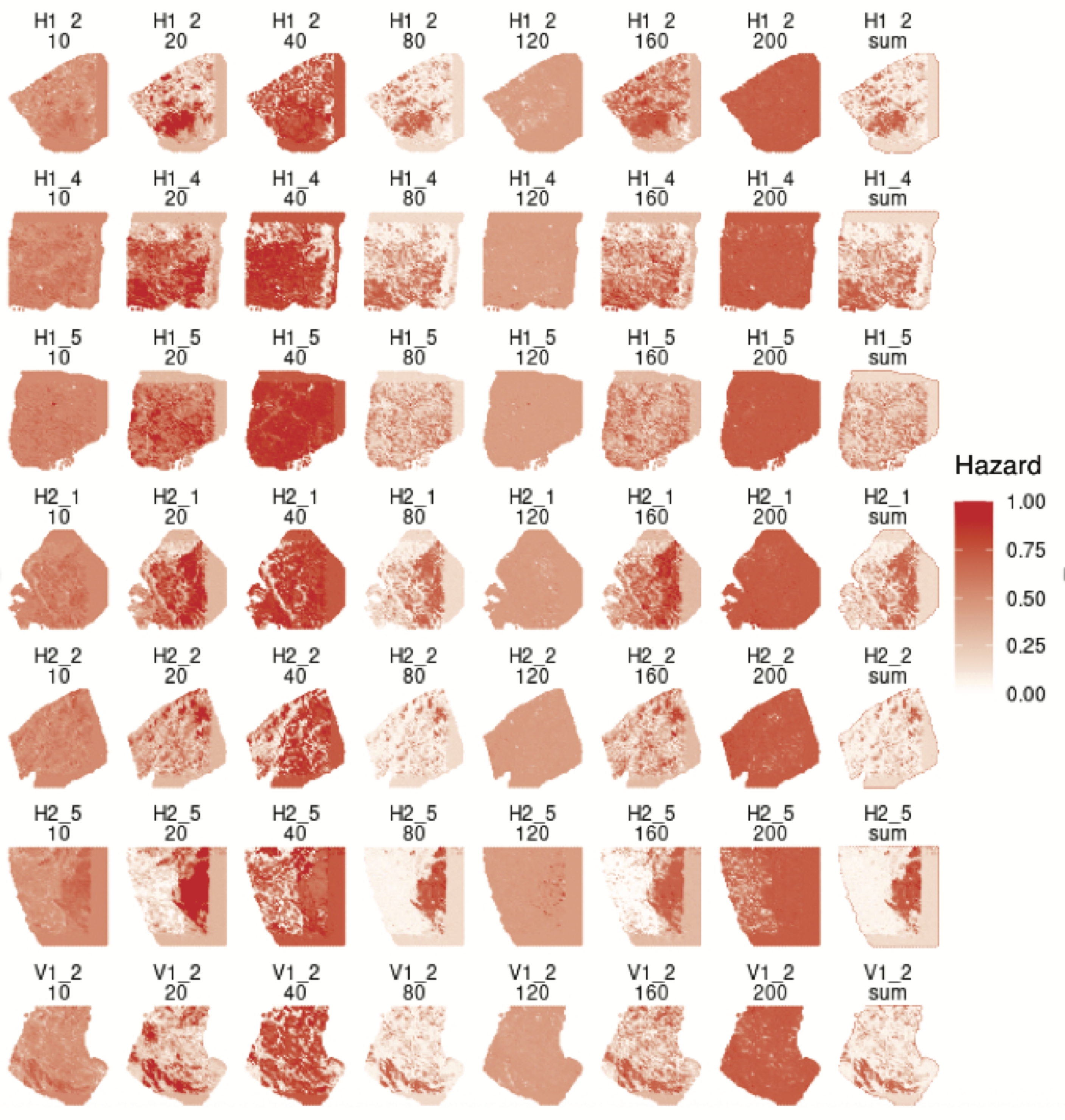

Supplement: qzaf119_Supplementary_Data [file qzaf119_supplementary_data.zip › Figure S2.tif]

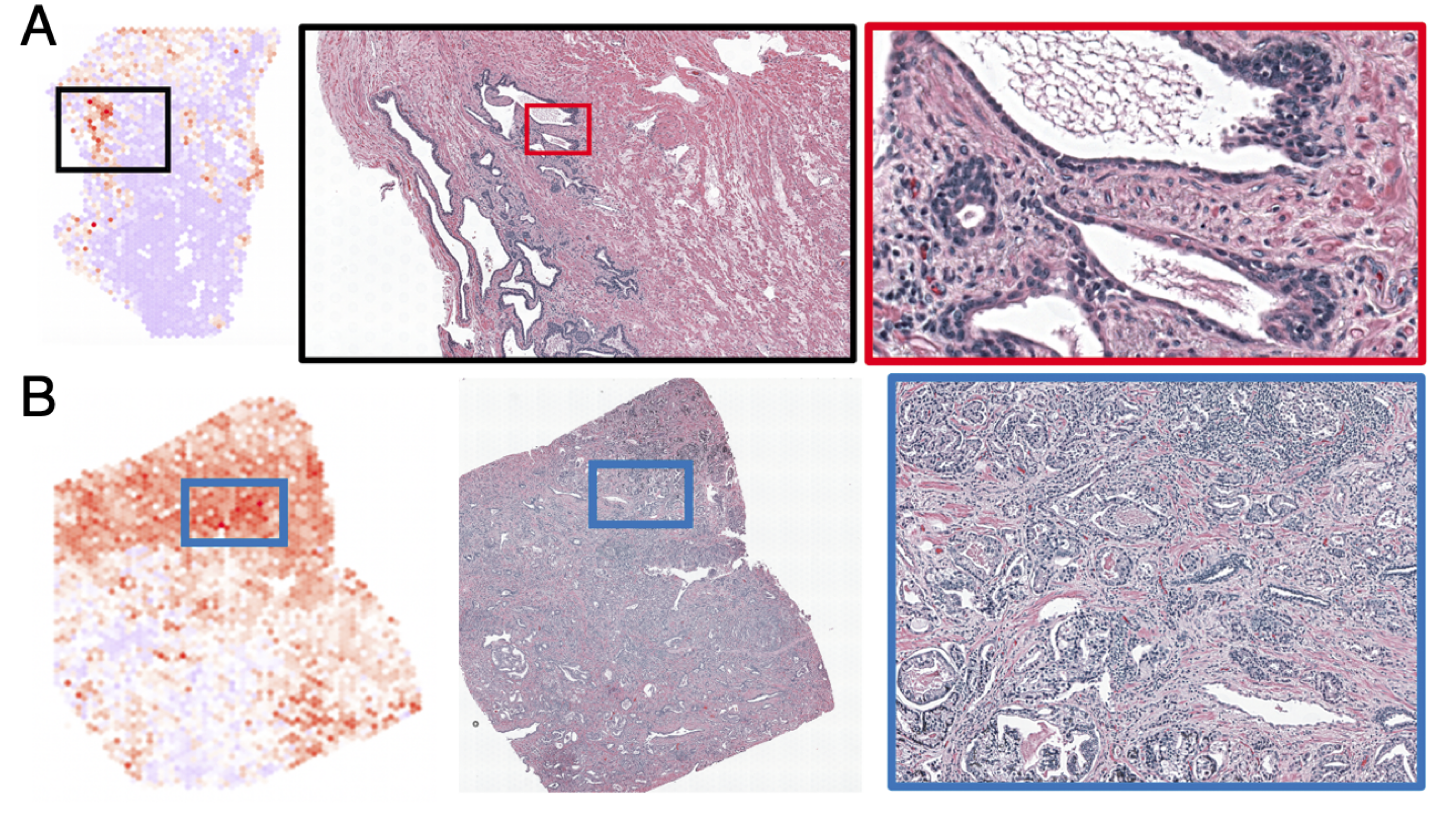

Supplement: qzaf119_Supplementary_Data [file qzaf119_supplementary_data.zip › Figure S3.tif]

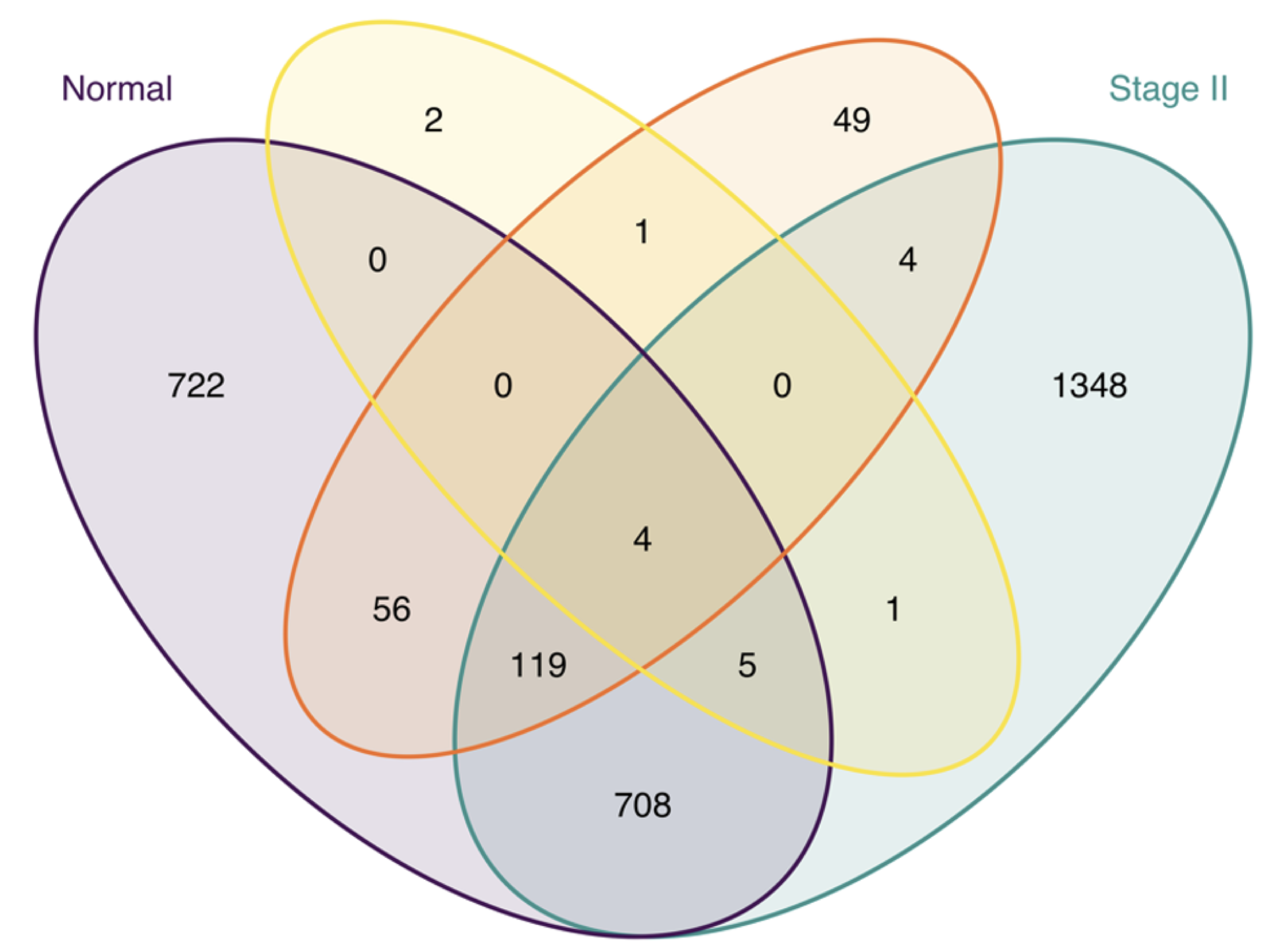

Supplement: qzaf119_Supplementary_Data [file qzaf119_supplementary_data.zip › Figure S4.tif]

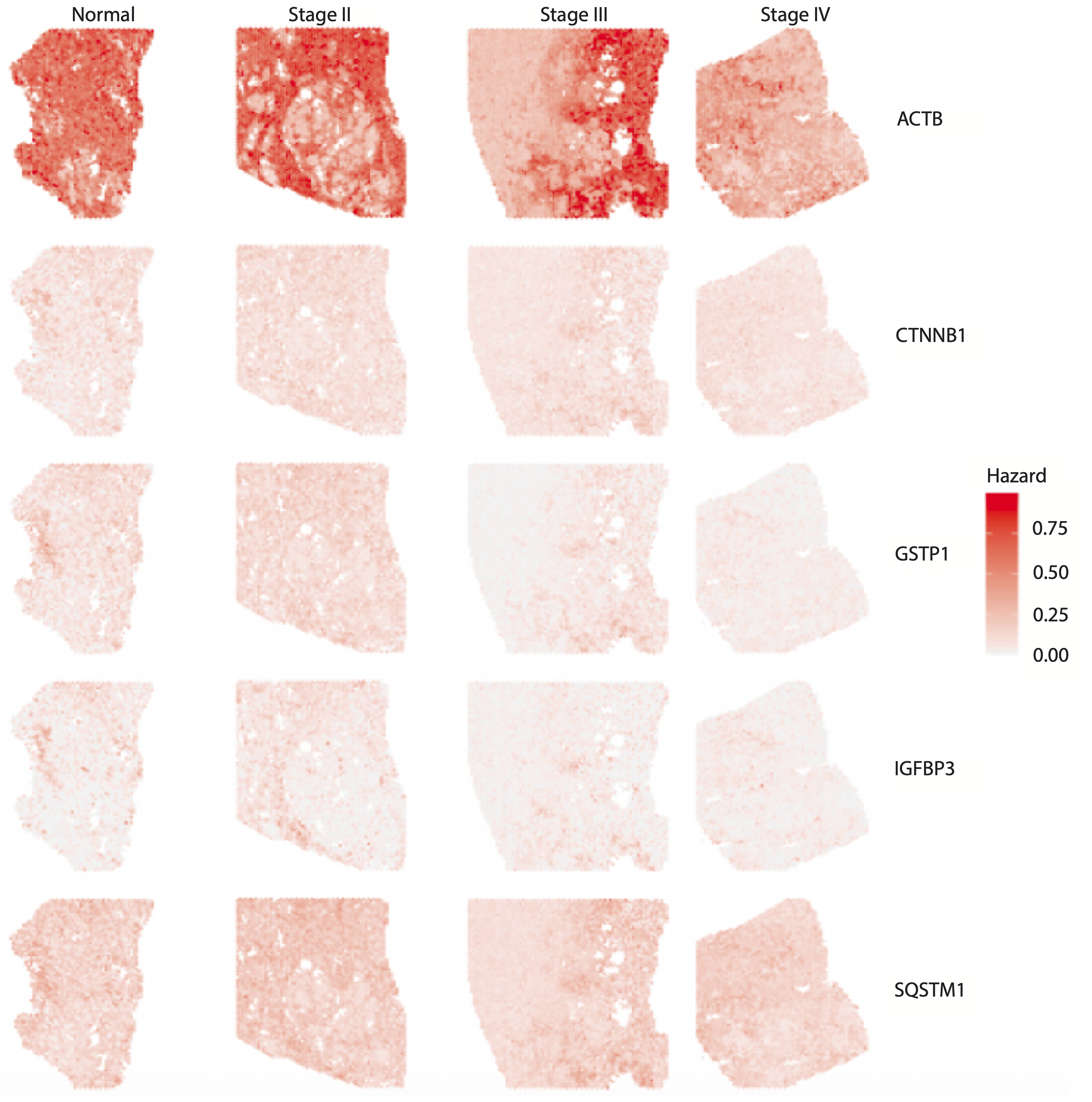

Supplement: qzaf119_Supplementary_Data [file qzaf119_supplementary_data.zip › Figure S5.tif]

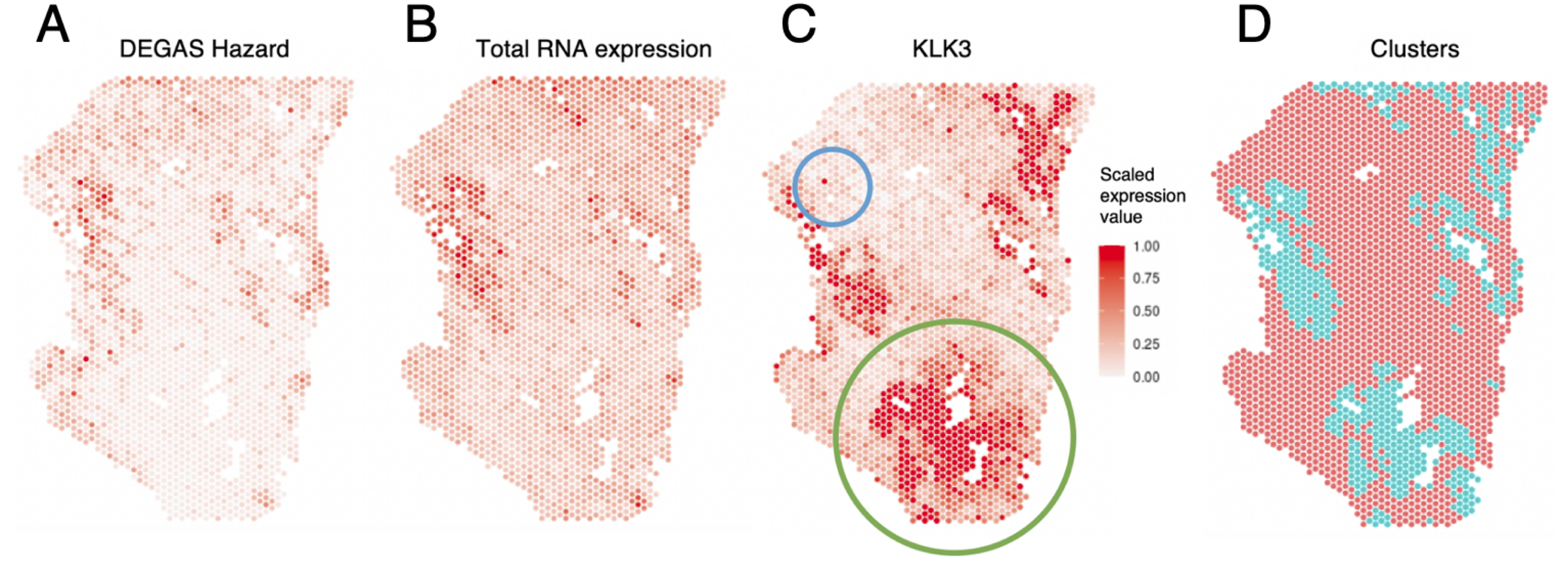

Supplement: qzaf119_Supplementary_Data [file qzaf119_supplementary_data.zip › Figure S6.tif]

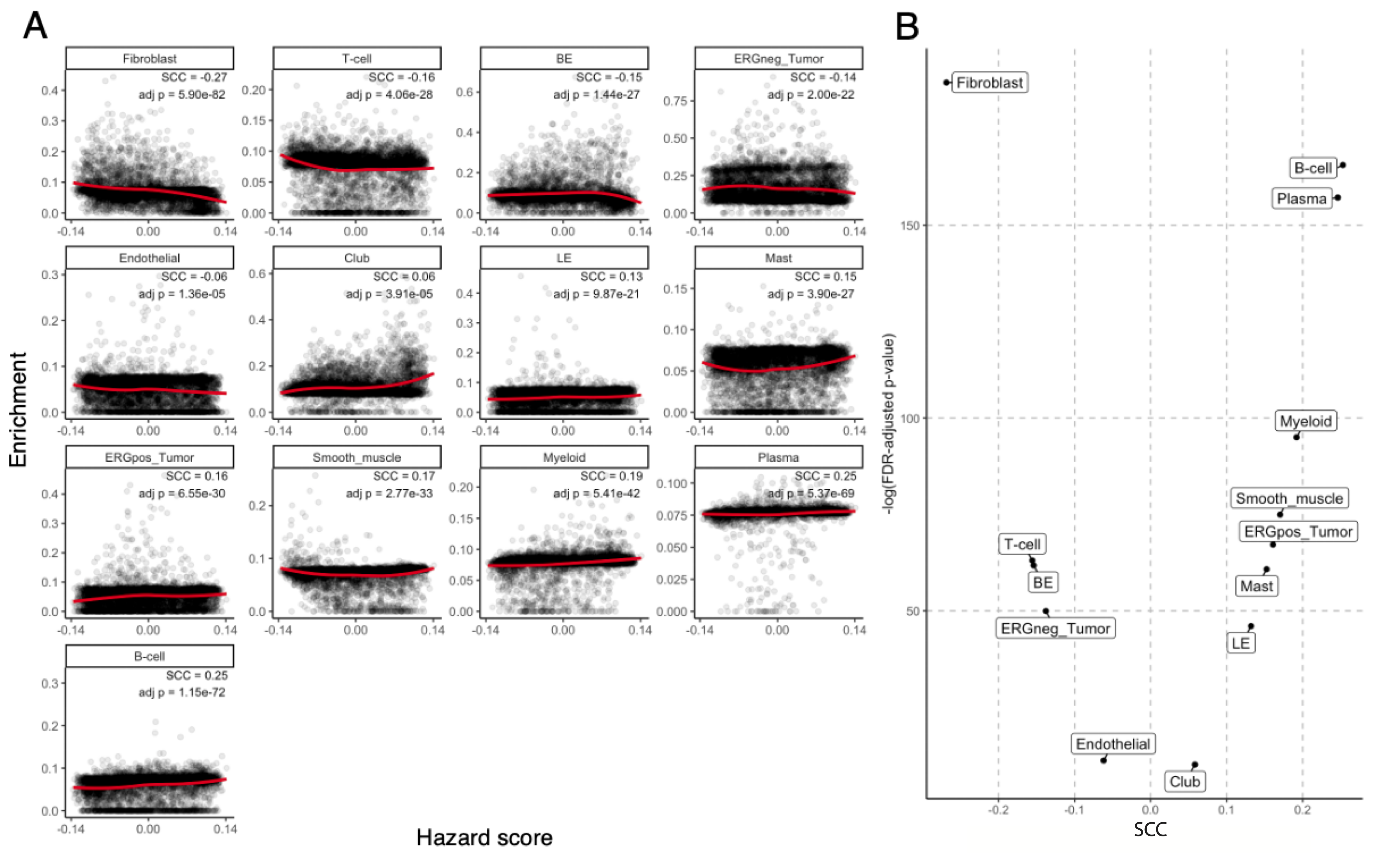

Supplement: qzaf119_Supplementary_Data [file qzaf119_supplementary_data.zip › Figure S7.tif]

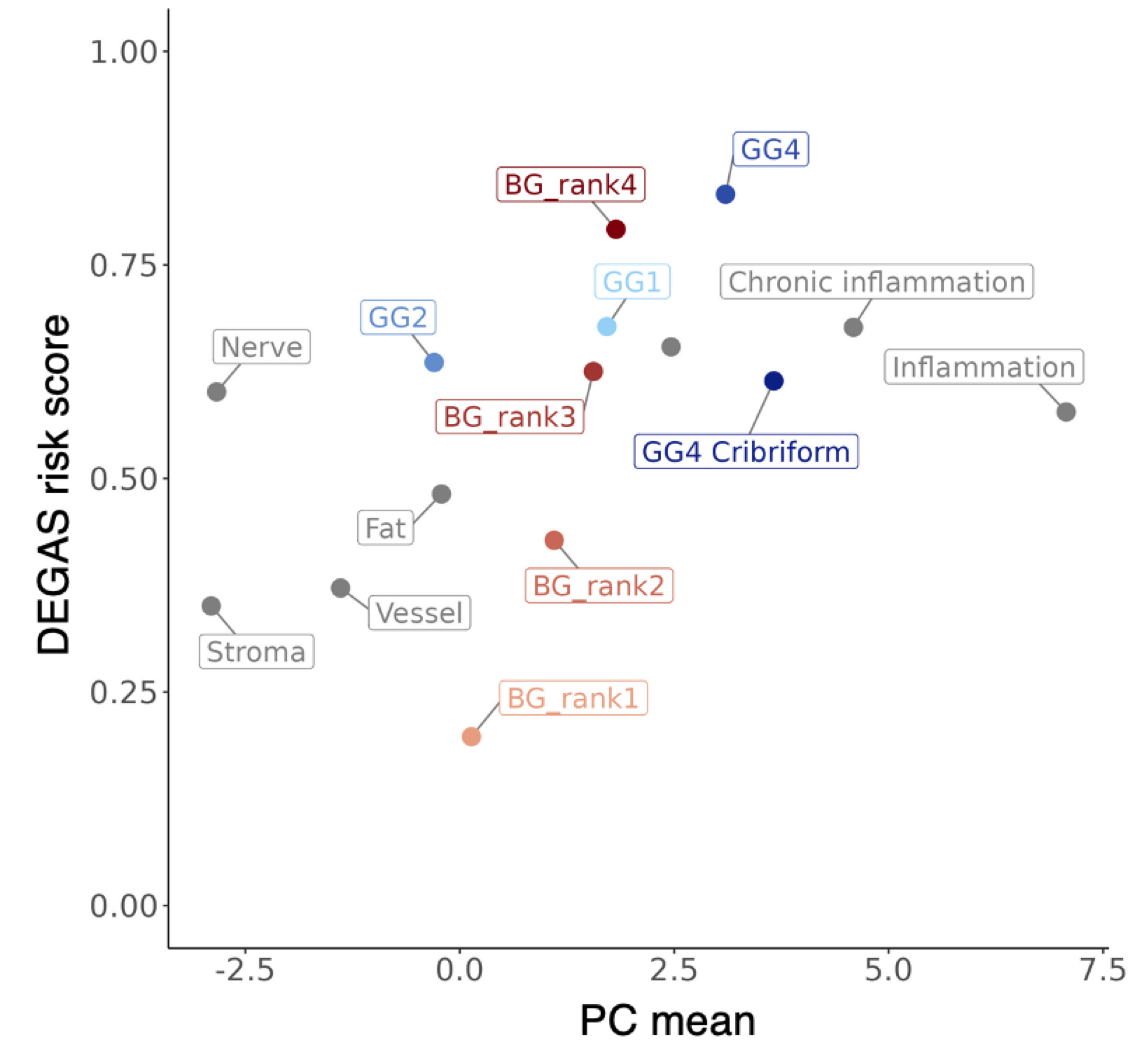

Supplement: qzaf119_Supplementary_Data [file qzaf119_supplementary_data.zip › Figure S8.tif]
